# A Simple, Ultrastable, and Cost‐Effective Oxygen‐Scavenging System for Long‐Term DNA‐PAINT Imaging

**DOI:** 10.1002/smll.202509092

**Published:** 2025-12-19

**Authors:** Rebecca T. Perelman, George M. Church, Johannes Stein

**Affiliations:** ^1^ Wyss Institute for Biologically Inspired Engineering Harvard University Boston Massachusetts USA; ^2^ Harvard Biophysics Program Harvard University Boston Massachusetts USA; ^3^ Department of Genetics Harvard Medical School Boston Massachusetts USA; ^4^ Max Planck Institute For Molecular Genetics Berlin Germany

**Keywords:** DNA‐PAINT, oxygen‐scavenger, single‐molecule, super‐resolution microscopy

## Abstract

DNA‐PAINT (Points Accumulation in Nanoscale Topography) is a super‐resolution microscopy technique capable of nanoscale imaging through the transient binding of fluorescently labeled imager strands to complementary DNA docking strands. Imager strands can be continuously replenished from an effectively infinite pool, making DNA‐PAINT inherently resistant to photobleaching. However, extended DNA‐PAINT imaging is limited by the formation of reactive oxygen species (ROS), which damage docking strands and reduce localization sampling over time. Although the state‐of‐the‐art oxygen‐scavenging system (OSS) can mitigate this damage, its enzymatic components degrade over time, reducing its performance, robustness, and utility. Here, we introduce a simple, enzyme‐free oxygen scavenging buffer based on sodium sulfite (Na_2_SO_3_) that overcomes these challenges. Our optimized formulation, combining Na_2_SO_3_ with Trolox (SST), effectively preserves docking strand integrity for over 24 h and enhances long‐term imaging performance. SST improves buffer stability tenfold, is easy to prepare and reduces costs by more than 90%, providing a robust, cost‐effective, high‐performance OSS for extended DNA‐PAINT imaging.

## Graphical abstract

A simple, enzyme‐free, and cost‐effective oxygen scavenging system enhances DNA‐PAINT super‐resolution microscopy by increasing photostability, minimizing docking strand degradation, and enabling faster, high‐precision localizations. Its long‐term stability supports extended imaging, yielding improved spatial resolution and image quality. This robust system provides a foundation for advancing a wide range of DNA‐based multiplexed imaging techniques.

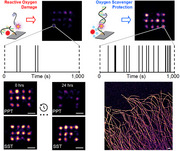

## 1 Introduction

Super‐resolution microscopy has become a powerful tool for visualizing biological structures at spatial resolutions beyond the diffraction limit of light [1, 2, 3, 4, 5]. Single‐molecule localization microscopy [6] (SMLM) achieves sub‐diffraction spatial resolution by temporally separating fluorescence emission through stochastic blinking. While highly effective, SMLM techniques can be limited by the irreversible loss of fluorescence due to photochemical damage during excitation, a phenomenon known as photobleaching. Photobleaching prevents repeated sampling of molecular locations and ultimately limits achievable resolution. DNA‐PAINT, which achieves nanometer‐precision localization through the transient binding of fluorescently labeled imager strands that produce stochastic blinking, offers resistance to photobleaching by continuously replenishing fresh imager strands from a large diffusing pool [7, 8]. This reversible binding mechanism provides continuous resampling and theoretically unlimited localizations, enabling extended imaging [9], molecular counting [10, 11], and resolution down to the level of single proteins [12, 13].

**Figure 1:**
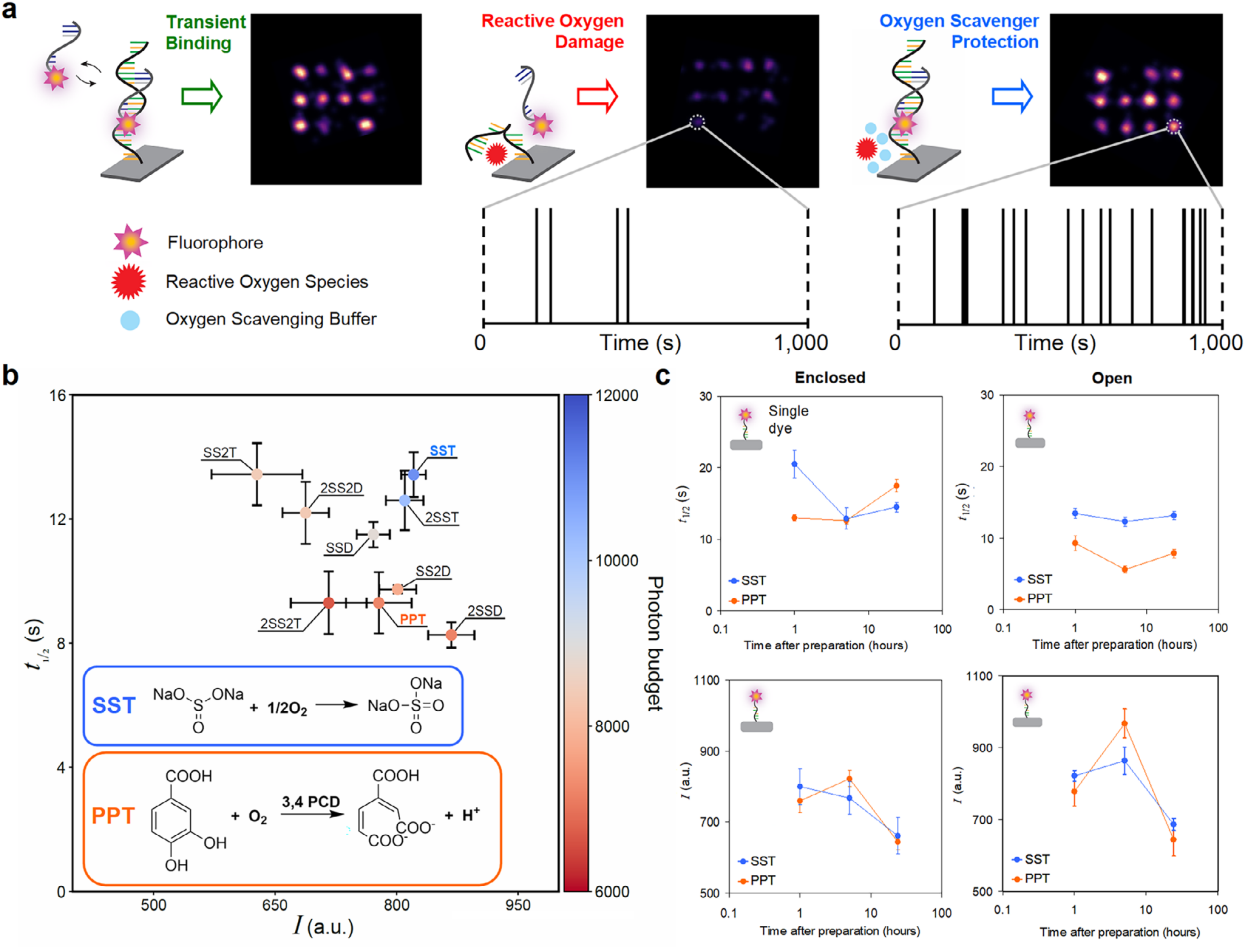
Stability and efficiency of DNA‐PAINT oxygen‐scavenging systems. (a) Schematic illustration of transient imager‐docking strand binding and ROS‐induced damage. The protective effects of oxygen scavengers are demonstrated by the number of localizations in super‐resolved images of a 20‐nm DNA origami grid. In the absence of scavengers, ROS damage leads to incomplete sampling of docking strands. In their presence, docking strands remain fully accessible, yielding efficient localization sampling and continuous imager binding over time. Experimental images shown within the schematic were acquired in a standard oxygen‐scavenging buffer (PPT, left), a buffer lacking an oxygen‐scavenger (Buffer C, middle), and the optimized oxygen‐scavenging buffer developed in this study (SST, right). All imaging was performed at the same imager concentration (∼235 pM), illumination (10 mW), magnification (100×), exposure time (200 ms), and images were rendered using the same parameters. (b) Measured fluorescence half‐life, *t*_1/2_, vs. average number of photons detected per localization, *I*. The corresponding photon budget (defined as *t*_1/2_ × *I*) is represented by the color scale indicated in the bar on the right. Conditions include SST, SSD, and PPT, as well as modified variants: 2SST and 2SSD (with doubled Na_2_SO_3_ concentrations), SS2T and SS2D (with doubled triplet state quenchers), and 2SS2T and 2SS2D (with both Na_2_SO_3_ and triplet state quenchers doubled). Inset: oxygen scavenging mechanisms for Na_2_SO_3_ and PCD. c), Fluorescence half‐life, *t*_1/2_ (top) and average number of photons detected per localization, *I* (bottom) measured as soon as possible after preparation and at 5 and 24 h thereafter for PPT (orange) and SST (blue) using single‐dye origami. Time after preparation is plotted on a logarithmic scale. Measurements were conducted in an enclosed chamber (left panel) and open chamber (right panel). Data are presented as means ±SEM (*n* ≥3 per condition).

However, irreversible photo‐induced damage to docking strands by reactive oxygen species (ROS) remains a limitation in DNA‐PAINT, restricting imaging durations and affecting both achievable resolution and quantitative interpretations [14]. Oxygen scavenging systems (OSSs) are widely used in SMLM to mitigate photobleaching by removing molecular oxygen, thereby extending fluorophore lifetime [6, 14, 15]. In DNA‐PAINT, OSSs also protect docking strands by limiting ROS generation [14] (Figure 1a). The state‐of‐the‐art DNA‐PAINT OSS [8, 16], denoted as ‘PPT’, contains protocatechuic acid (PCA), protocatechuate‐3,4‐dioxygenase (PCD), and the triplet state quencher Trolox. PPT maintains optimal photophysical conditions and effectively protects docking strands for up to ∼1–2 h [17]. However, in the presence of oxygen, the PCD‐driven reaction gradually acidifies the buffer (Figure 1b, inset), reducing scavenging efficiency [17] and altering DNA binding dynamics [18]. As a result, frequent buffer replacement is required, posing challenges for high‐throughput or multiplexed DNA‐PAINT workflows that require tens of sequential imaging rounds [13, 19, 20] or for fluidics‐based automation [21]. In addition, PPT preparation is labor‐intensive and costly, limiting its scalability.

Sodium sulfite (Na_2_SO_3_), a chemical oxygen scavenger (Figure 1b, inset), has recently been applied in the context of fluorogenic DNA‐PAINT [22], where ROS formation is intrinsically reduced as quencher‐labeled imager strands are protected from photobleaching during diffusion [23]. However, its potential as standalone OSS for conventional DNA‐PAINT remains unexplored, and a quantitative comparison with PPT is missing. Given its success in stabilizing imaging buffers for the SMLM variant STORM [24, 25, 26] (Stochastic Optical Reconstruction Microscopy), Na_2_SO_3_ presents a promising, simpler, and more cost‐effective alternative that warrants systematic assessment.

We systematically evaluated Na_2_SO_3_‐based buffer formulations for their performance in DNA‐PAINT imaging. We screened various Na_2_SO_3_ concentrations with the triplet‐state quenchers Trolox or DABCO [25, 26] in single‐dye experiments and identified Na_2_SO_3_ and Trolox (SST) as the optimal formulation for conventional DNA‐PAINT. SST showed 1.4‐ to 2.2‐fold greater photostability than PPT and preserved docking strand integrity over 24 h, resulting in a tenfold improvement in buffer stability and ∼90% reduction in cost, with SST costing ∼$0.02 mL^−1^ (primarily due to Trolox) compared to ∼$0.31 mL^−1^ for PPT. SST enables long‐term DNA‐PAINT imaging by minimizing ROS formation and preserving docking strand integrity. It remains stable at room temperature for weeks, supports extended acquisitions without compromising image quality or the imager binding frequency, thereby improving the spatial resolution through enhanced sampling density [6, 27].

## 2 Results

### 2.1 Long‐Term Photostability of Oxygen‐Scavenging Buffers in Single‐Dye DNA Origami Imaging

To evaluate Na_2_SO_3_ performance in combination with different triplet‐state quenchers, we started with 30 mm Na_2_SO_3_, the optimal concentration identified for STORM imaging [25], and formulated two buffers: one with 1 mM Trolox (SST), as used in PPT [8], and the other with 65 mM DABCO [25] (SSD). We also prepared variants with doubled concentrations of both Na_2_SO_3_ and triplet‐state quenchers. Photostability was assessed using Cy3B‐labeled single‐dye DNA origami structures [28, 29], the most robust fluorophore for 560 nm excitation in DNA‐PAINT [30] (Figure S1a). We measured the fluorescence half‐life, *t*_1/2_, defined as the time at which half the dyes were photobleached upon continuous illumination, and the average number of photons detected per localization, *I*. Consistent with previous reports [29], single Cy3b dyes bleached on the timescale of tens of seconds. SST showed the best performance, with a 40% in *t*_1/2_ (13.4 s vs. 9.3 s) and 50% increase in photon budget (∼12 000 vs. 8000) compared to PPT (Figure 1b).

Next, we evaluated SST performance and stability over 24 h compared to PPT, using single‐dye DNA origami in enclosed and open chambers to control oxygen exchange. In the enclosed chamber, which prevented oxygen influx, both buffers performed similarly (Figure 1c, left). We note that *t*_1/2_ decreased after 5 h in the SST condition. This effect is likely due to the use of tape and epoxy in the chamber construction, which can trap and gradually release residual oxygen, leading to localized retention. Since SST's scavenging efficiency depends directly on oxygen concentration, this transient oxygen availability may alter bleaching kinetics and could explain the observed decrease in *t*_1/2_ in the enclosed chamber. In the open chamber, where oxygen exchange was permitted and representative of typical DNA‐PAINT conditions [9], the *t_1/2_* of PPT declined over the 24‐h period, dropping by 40% within 5 h of preparation, while SST showed only a 10% decline, with values comparable to its performance in the sealed chamber (Figure 1c, right). After 5 h, the *t_1/2_* in SST was more than twice that of PPT, demonstrating its superior photostability and long‐term performance.

### 2.2 Characterization of DNA‐PAINT Docking Strand Stability Over Time

To assess whether SST minimizes photoinduced docking strand damage, we employed a DNA origami structure featuring twelve docking strands spaced 20 nm apart [8] (Figure S1b). As in previous work [14], we conducted extended DNA‐PAINT acquisitions of 25 000 frames at 200 ms exposure (∼1.5 h), during which freshly prepared PPT is known to preserve docking strands [14]. Since ∼5000 frames are sufficient to localize all sites, we compared super‐resolved images from the initial and final 5000 frames (referred to as ‘first segment’ vs. ‘last segment’, hereafter) to quantify photoinduced damage and imaging fidelity [14].

**Figure 2:**
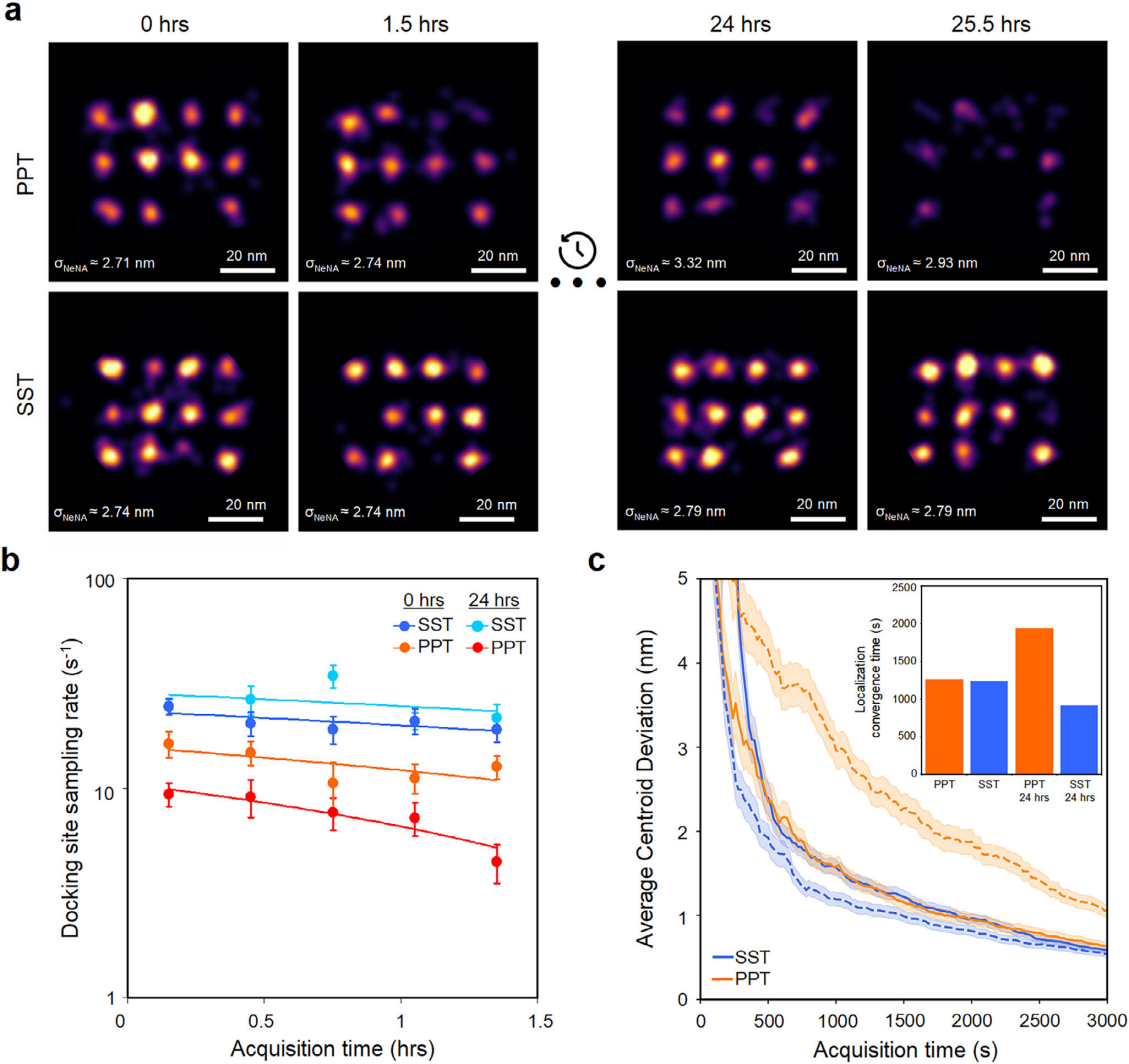
SST enhances DNA‐PAINT docking strand preservation, resolution, and sampling density, as evaluated by extended imaging of DNA origami and analysis of damage rates. (a) Representative DNA origami images acquired in PPT and SST (top and bottom, respectively) over 25.5 h, including two 1.5‐h imaging sessions separated by 24 h of idle time. In PPT, image intensity and quality progressively declined, whereas in the SST, they remained stable. The σ_NeNA_ localization precision is stated in each image. All imaging was performed at the same imager concentration, illumination (10 mW), magnification (100×), exposure time (200 ms), and images were rendered using the same parameters. Scale bars: 20 nm. (b) Docking strand sampling rate in both buffers on logarithmic scale. While both buffers showed a similar decline in docking strand sampling rate during the first 1.5‐h imaging session, the rate in PPT continued to decline over the 24‐h idle period, whereas it fully recovered in SST. In the second 1.5‐h session, the rate remained stable in SST but dropped significantly in PPT. Data are presented as means ± SEM (*n*=11). Linear fits on the log scale indicate exponential decay. (c) Convergence behavior of RESI center localizations relative to the full 25 000‐frame dataset. Both buffers exhibited similar convergence rates immediately after preparation (solid lines), whereas SST showed markedly faster convergence than PPT after 24 h (dashed lines). Data are presented as means ± SEM (*n*=100).

To evaluate the long‐term stability of OSS performance, we conducted two rounds of extended DNA‐PAINT acquisitions for each condition (PPT and SST): one immediately after OSS preparation and another 24 h after OSS preparation. Between experiments, samples were stored at room temperature in their respective OSSs. All imaging conditions, including DNA origami and imager strand concentrations, as well as excitation laser power, were held constant. Super‐resolved DNA origami images were reconstructed using identical parameters (Figure 2a). Localization precision (*σ*_NeNA_) for each dataset was quantified using the nearest‐neighbor analysis (NeNA) method [31], which estimates the localization uncertainty at individual, spatially defined docking strands. Immediately after preparation, both OSSs yielded comparable image quality and *σ*_NeNA_ values in the first segment: 2.74 nm for SST and 2.71 nm for PPT (Figure 2a, top left). After 1.5 h of imaging, as expected, only minor signs of deterioration were observed in PPT, including reduced localization density and occasional docking strand loss in the last segment. In contrast, SST maintained image integrity without detectable deterioration (Figure 2a, bottom left. See Figure S2 for additional 100 exemplary DNA origami per OSS).

After 24 h of storage, the performance differences between both OSSs became more pronounced. When extended DNA‐PAINT acquisitions were performed at a new field of view, PPT exhibited a significant decline in image quality as early as in the first segment. This was evident in reduced localization sampling and increased localization uncertainty (*σ*_NeNA_ = 3.32 nm), and clear signs of damage to several docking strands. Over the following 1.5 h, degradation continued, and by the last segment, many docking strands were barely detectable (Figure 2a, top right). In striking contrast, SST preserved high localization precision (∼2.79 nm) and dense localization sampling throughout the entire acquisition, with minimal apparent docking strand damage even after prolonged imaging (Figure 2a, bottom right. See Figure S3 for additional 100 exemplary DNA origami per OSS).

To further quantify these differences, we measured the number of binding events per docking strand, defined as the docking strand sampling rate (s^−1^), in both the first and last segments of the datasets immediately after buffer preparation and again after 24 h. To ensure reproducibility, sampling rate measurements were replicated across three independently prepared samples (Figure S4a). Notably, SST supported nearly a twofold higher docking strand sampling rate compared to PPT in the initial segments (∼17 s^−1^ vs. ∼9 s^−1^), despite identical imager strand concentrations. This suggests that SST not only preserves docking strand integrity over time but also substantially enhances hybridization kinetics.

Following preparation, both OSSs exhibited a comparable reduction in active docking strands between the first and last segments (20% decrease in SST vs. 23% in PPT). However, after 24 h of storage, docking strand activity continued to decline in PPT, whereas it fully recovered in SST, consistent with trends observed in Figure 2a. During the second 1.5‐h imaging session, the docking strand sampling rate remained stable in SST (13% decrease), while a marked decline was measured in PPT (52% decrease) (Figure 2b). We further evaluated both OSSs one month after preparation. Strikingly, SST maintained high performance with only a 10% decrease in sampling rate over the 1.5‐h imaging round, while PPT showed substantial degradation, marked by a 54% decrease (Figure S4b). These findings suggest that preparing larger buffer stocks for extended use may be advantageous, although we note that the one‐month storage experiment was performed only once.

### 2.3 SST Improves RESI Convergence and Preserves Exchange‐PAINT Orthogonality

Next, we examined the effects of each OSS on additional technical aspects of DNA‐PAINT imaging. A recent advancement, RESI (Resolution Enhancement by Sequential Imaging), introduced by Reinhardt et al., offers a powerful approach to enhance localization precision [13]. RESI builds on the concept of performing Exchange‐PAINT [9] on a single target species to generate sparse localization clouds, each corresponding to an individual molecule. By precisely determining the center of these ‘single‐docking strand’ clouds, the method enables pinpointing docking strand positions with reported precision down to the Ångström scale [13]. Due to their sequential nature, both RESI and Exchange‐PAINT require docking strands to remain intact over extended imaging times, exceeding those typical of conventional DNA‐PAINT. In this context, we sought to assess the compatibility of SST with such demanding imaging protocols.

**Figure 3:**
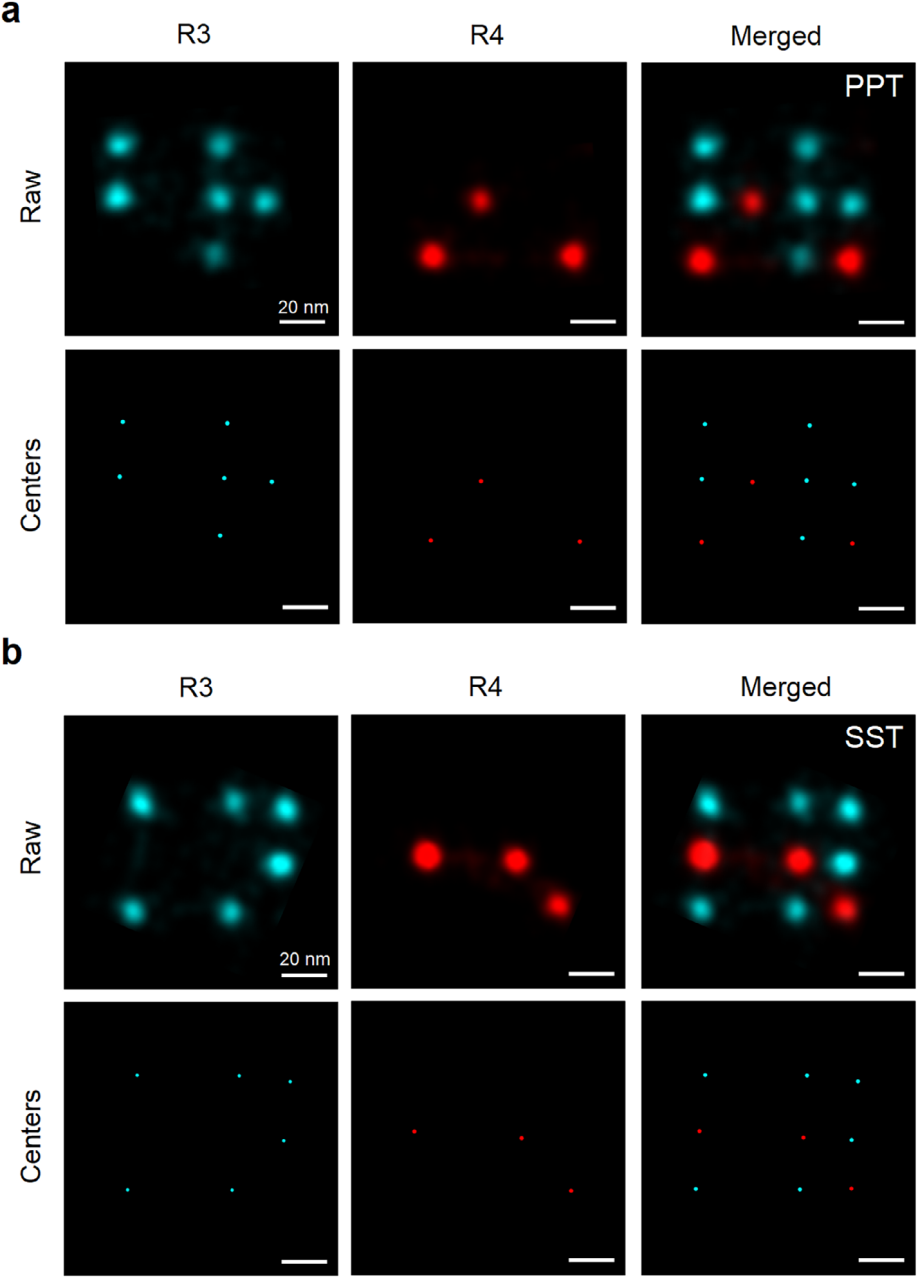
Exchange‐PAINT origami images and RESI analysis. (a,b) Representative origami images from Exchange‐PAINT experiments in PPT (a) and SST (b) showing individual exchange rounds for 7×ACA (red) and 7×CTC (cyan). Raw localizations (“Raw”) and corresponding cluster centers (“Centers”) are shown for each channel, along with a merged view. Scale bar: 20 nm. All imaging was performed at the same imager concentration (∼1 nM), illumination (10 mW), magnification (100×), exposure time (200 ms), and images were rendered using the same parameters.

Given that our DNA origami design enables single‐docking‐strand resolution, we leveraged our extended DNA‐PAINT datasets to assess performance in the context of RESI analysis. Briefly, RESI localizations were calculated by determining the weighted mean of the x‐ and y‐coordinates from all localizations within a given localization cloud. For each of the four conditions (PPT fresh, PPT after 24 h, SST fresh, and SST after 24 h) we randomly selected 100 individual docking strands and computed their RESI center localizations from the full 25 000‐frame dataset, serving as our ground‐truth reference. To evaluate convergence behavior, we analyzed subsets of the data at shorter acquisition durations, quantified the deviation of each RESI localization from the full‐data estimate, and determined the imaging time required for RESI localizations to converge to that reference. As expected, both SST and PPT performed comparably immediately after preparation, requiring approximately 21 min of imaging to achieve an e‐fold improvement in localization precision. However, after 24 h, RESI convergence in PPT was markedly impaired, requiring up to 60% longer acquisition times to achieve comparable accuracy, highlighting a clear decline in performance. In contrast, SST not only preserved but even improved its performance after 24 h, reaching the same precision threshold in just 15 min compared to 21 min previously, further underscoring its suitability for RESI applications (Figure 2c). We further confirmed that SST preserves the orthogonality of docking and imager strands by performing sequential Exchange‐PAINT imaging [9]. Using a DNA origami structure containing a stochastic mixture of two orthogonal speed docking sequences [32, 33] (R3 and R4), no cross‐talk between the sequences was observed in either PPT or SST buffers (Figure S5). RESI images reconstructed from the Exchange‐PAINT datasets demonstrated that SST supports the robust orthogonality necessary for RESI (Figure 3). Overall, these results show that SST not only protects docking strands from damage but also preserves consistent localization sampling, enabling reliable high‐precision imaging over extended acquisition times.

For unknown biological structures, the positions of docking strands are not known in advance. Therefore, achieving high a localization density is essential to ensure that structural features are adequately resolved. According to the Nyquist‐Shannon sampling criterion [34], resolution is fundamentally limited to twice the average spacing between localizations, meaning that a reduction in available docking strands over time directly constrains the highest resolvable spatial frequencies [27]. In Supplementary Note S1 we discuss the theoretical implications on resolution loss on unknown structures based on the docking strand damage rates obtained from DNA origami experiments (Figure S6a,b).

### 2.4 Characterization of SST Performance in Cells

**Figure 4:**
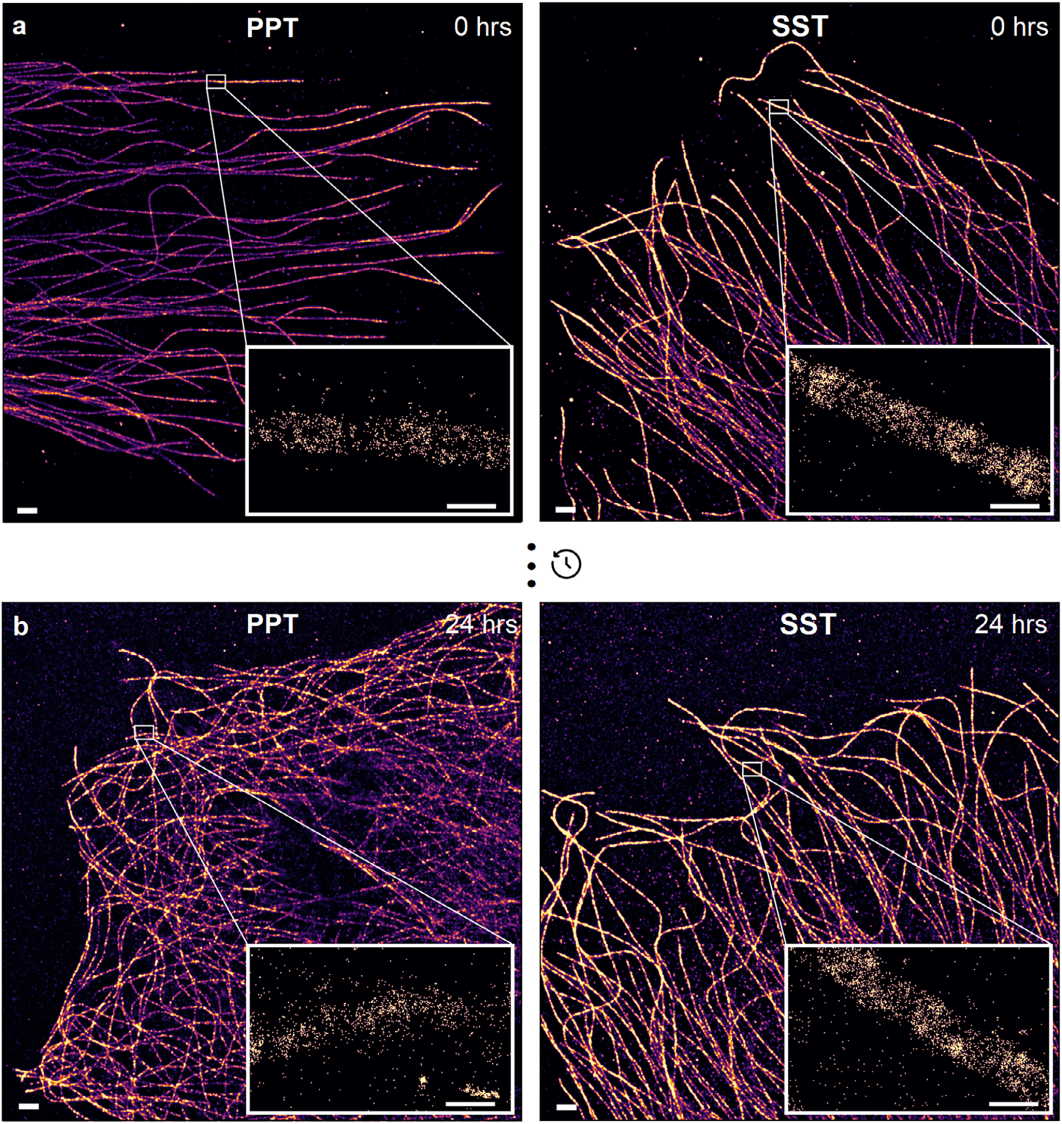
The advantages of SST extend to DNA‐PAINT imaging in fixed cells. (a) DNA‐PAINT images of the microtubule network in fixed HeLa cells acquired in PPT (left) and SST (right), with *σ*_NeNA_=3.7 nm in both conditions. Images were acquired at the same imager concentration, illumination (10 mW), magnification (100×), exposure time (200 ms), and displayed using the same intensity range. Inset: zoom‐in views range rendered at the same parameters. (b) DNA‐PAINT images of the microtubule network in fixed HeLa cells acquired in PPT (left) and SST (right) 24 h after preparation, with *σ*_NeNA_ values of 4.7 and 4.3 nm, respectively. Images were acquired under the same conditions. Inset: zoom‐in views rendered at the same parameters. Scale bars: 1000 nm. Scale bars: 100 nm. FOV: 130 × 130 µm^2^.

While DNA origami serves as an excellent benchmark for assessing docking strand damage in DNA‐PAINT experiments, it does not fully reflect the complexity of cellular imaging. To assess the suitability of SST for cellular experiments and to compare overall image quality, we performed extended DNA‐PAINT acquisitions targeting microtubule filaments in fixed HeLa cells. Imaging was carried out using both PPT and SST, immediately after buffer preparation and again after a 24‐h storage period. As before, each acquisition comprised 25 000 frames, allowing comparison between the initial and final 5000‐frame segments. Figure 4a shows the super‐resolved microtubule reconstructions for both OSSs over the full acquisition period immediately after preparation and demonstrates that SST performs at least as well as PPT in cellular imaging applications with *σ*_NeNA_ values of 3.7 nm in both conditions. Notably, a substantially lower imager concentration was used in the microtubule experiments compared to the DNA origami assays. Since ROS‐induced docking strand damage is concentration‐dependent [14], the performance difference between SST and PPT was less pronounced in microtubule imaging. Only a minor reduction in localization precision was observed for both conditions (4.3 nm for SST vs. 4.7 nm, for PPT), likely reflecting sample‐related factors such as antibody unbinding during overnight storage. Nevertheless, consistent with DNA origami results, SST yielded a significantly higher docking strand localization density than PPT in cellular imaging (Figure 4, Figure S7a).

This effect was further quantified by measuring the docking strand sampling rate across microtubule segments (Figure S7b). PPT showed a ∼30% decline between sessions, compared to ∼10% in SST. Moreover, SST yielded over twice the localization density of PPT, highlighting its improved retention of docking strand activity and imaging performance. To assess structural preservation, cross‐sectional fluorescence intensity profiles of microtubules imaged in PPT and SST at 0 and 24 h were analyzed. All profiles reflected the expected hollow, rod‐like morphology with peak‐to‐peak distances of ∼38 nm, consistent with reported values [33, 35] (Figures S8 and S9). These findings demonstrate that SST is fully compatible with cellular DNA‐PAINT imaging and that its in vitro performance improvements translate robustly to cellular contexts.

## 3 Conclusion

In summary, we present SST as an ultra‐stable and highly efficient oxygen‐scavenging system for conventional DNA‐PAINT and Exchange‐PAINT imaging that significantly enhances image quality, extends imaging durations, and improves resolution by increasing photostability and minimizing docking strand damage. While alternative strategies exist for reducing molecular oxygen, including vacuum systems, DNA‐mediated delivery of photostabilizers [36], and fluorogenic dyes that resist photobleaching [22], these approaches tend to introduce additional complexity and cost. In contrast, SST is simple, cost‐effective, and easy to implement using standard laboratory reagents.

The advantages of SST demonstrated here are based on its compatibility with Cy3B, a high‐performance orange fluorophore widely used in DNA‐PAINT [8, 13, 19, 20, 30]. By chemically removing dissolved oxygen, SST creates a low oxygen environment in which bleaching kinetics are primarily governed by oxygen availability. Accordingly, while fluorophore lifetimes vary by dye, the enhanced stability observed with Cy3B is expected to extend to other Trolox‐compatible dyes under open chamber DNA‐PAINT conditions [30]. Recently introduced ‘ANice’, a non‐toxic and cost‐effective OSS optimized for green fluorophores [37], indicates a broader push toward broadly‐accessible imaging buffers. Although no universal OSS exists, comprehensive fluorophore screens provide a foundation for adapting SST to additional dyes [30]. This is particularly important for multicolor DNA‐PAINT applications requiring spectral demixing [38].

Beyond DNA‐PAINT, a major advantage of SST lies in its ability to preserve DNA docking strands during prolonged imaging sessions—a critical requirement for a wide range of DNA‐based multiplexed techniques, such as Oligopaints/OligoSTORM [39, 40], MERFISH [41], seqFISH [42], SABER [43], OligoFISSEQ [44], DNA‐SOFI [45], or DNA‐STED [46] which rely on fixed DNA labels and barcoding strategies. By minimizing oxidative damage and maintaining the stability of DNA labels, SST has the potential to enhance the robustness and fidelity of these imaging strategies.

Together, our findings position SST as a versatile and accessible enhancement to DNA‐PAINT and as a strong foundation for DNA‐based multiplexed imaging.

## 4 Experimental Section/Methods

### 4.1 Materials

Unmodified, dye‐labeled, and modified DNA oligonucleotides were purchased from Integrated DNA Technologies, Metabion, and Biomers. Unmodified oligos were purified via standard desalting and modified oligos via HPLC. DNA scaffold strands were purchased from Tilibit (p7249, identical to M13mp18). Sample chambers were ordered from Ibidi GmbH (8‐well 80827 and 18‐well 81817). Tris 1 M pH 8.0 (AM9856), EDTA 0.5 M pH 8.0 (AM9261), Magnesium 1 M (AM9530G) and Sodium Chloride 5 M (AM9759) were ordered from Ambion. Streptavidin (S‐888) Ultrapure water (15568025), PBS (20012050), 4',6‐Diamidino‐2‐Phenylindole, Dihydrochloride (D1306) (A39255), BSA (AM2616), DMEM (10569), and Dithiothreitol (DTT) were purchased from Thermo Fisher Scientific. BSA‐Biotin (A8549), Tween‐20 (P9416‐50ML), Glycerol (cat. 65516‐500 mL), (+‐)‐6‐Hydroxy‐2,5,7,8‐tetra‐methylchromane‐2‐carboxylic acid (Trolox) (238813‐5G), methanol (32213‐2.5L), 3,4‐dihydroxybenzoic acid (PCA) (37580‐25G‐F), protocatechuate 3,4‐dioxygenase pseudomonas (PCD) (P8279‐25UN), sodium sulfite (S0505‐250G), 1,4‐Diazabicyclo[2.2.2]octane (DABCO) (D27802‐25G), Triton‐X 100 (93443), Sodium Azide (S2002), and HEPES (H4034‐100G) was purchased from Sigma‐Aldrich. 10% fetal bovine serum was purchased from Genesee Scientific (25‐514). EM grade gluyaraldehyde was purchased from Electron Microscopy Services (16220). 90 nm gold nanoparticles (G‐90‐20‐10 OD10) were purchased from Cytodiagnostics. Primary anti a‐tubulin (rabbit, 2125BF) antibody was purchased from Cell Signaling. Secondary goat anti‐mouse labeled with Alexa488 antibody (A11029) was purchased from Thermo Fisher Scientific. 0.5‐mL Amino Ultra Centrifugal Filters with 50 and 10 kDa molecular weight cutoffs were purchased from Millipore (UFC5050 and UFC5010, respectively). DBCO‐sulfo‐NHS ester cross‐linker was purchased from Vector Laboratories (CCT‐A124). Qubit Protein Assay (Q33211), NuPage 4%–12% Bis‐Tris protein gels (NP0323BOX), NuPage LDS Sample Buffer (NP0007) were purchased from Invitrogen. InstantBlue Coomassie Protein Stain was purchased from Abcam (ab119211).

### 4.2 Buffers

Four buffers were used for sample preparation and imaging: Buffer A (10 mM Tris‐HCl pH 7.5, 100 mM NaCl); Buffer B (5 mM Tris‐HCl pH 8.0, 10 mM MgCl2, 1 mM EDTA); Buffer C (1× PBS, 500 mM NaCl); 10× folding buffer (100 mM Tris,10 mM EDTA pH 8.0, 125 mM MgCl2). Antibody storage buffer: 1% BSA, 0.1% Sodium Azide, 10 mM EDTA, 50% glycerol). Buffers were checked for pH. For imaging, Buffer C was supplemented with oxygen scavenging & triplet state quenching system PPT (1× PCA, 1× PCD, 1× Trolox) or SST (30 mM Sodium Sulfite, 1× Trolox) prior to imaging.

### 4.3 PCA, PCD, Trolox

100× Trolox: 100 mg Trolox, 430 µL 100% Methanol, 345 µL 1 M NaOH in 3.2 mL H2O. 40× PCA: 154 mg PCA was mixed with 10 mL water adjusted to pH 9.0 with NaOH. 100× PCD: 9.3 mg PCD, 13.3 mL of buffer (100 mM Tris‐HCl pH 8, 50 mM KCl, 1 mM EDTA, 50% glycerol).

### 4.4 Sodium Sulfite, Trolox

100× Trolox: 100 mg Trolox, 430 µL 100% Methanol, 345 µL 1 M NaOH in 3.2 mL H2O. 1 M Sodium Sulfite: 1,764 mg Sodium Sulfite, 14 mL H_2_O, and can be left at room temperature for at least 1 month.

### 4.5 DNA Origami Design and Assembly

DNA origami structures were designed using the Picasso Design1 module. A list of all used DNA strands and their respective ordering conditions can be found in Tables S1–S3 We used two previously published DNA origami designs [8, 11, 29]: single dye (SD) and 20‐nm grid origami structures (Figure S1). The single dye origami structure had a single extension at the top side at position 2B07 of Picasso Design labeled permanently with a Cy3B molecule. The 20 nm grid was a 3 × 4 grid motif with 20‐nm spaced docking strands. Folding of structures was performed using the following components: single‐stranded DNA scaffold (0.01 µM), core staples (0.1 µM), biotin staples (0.01 µM), extended staples for DNA‐PAINT (each 1 µM), 1x folding buffer in a total of 50 µL for each sample. Annealing was done by cooling the mixture from 80°C to 25°C in 3 h in a thermocycler. Using a 1:1 ratio between scaffold and biotin staples allows sample preparation without prior DNA origami purification, where otherwise free biotinylated staples would saturate the streptavidin surface and prevent origami immobilization on the glass surface. For non‐Exchange‐PAINT experiments, the docking strand sequence consisted of a 17 nt motif (TT 5 × CTC), and as imager strand sequence we used a 7 nt motif (Pm2‐Cy3B), which directly hybridized to the docking strand sequence. For Exchange‐PAINT experiments, we used a 20 nt adapter motif “A20” incorporated into the same 20 nm grid DNA origami design, which allowed us to hybridize two docking strands via a stably binding complementary adapter (see Tables S3 and S4).

### 4.6 DNA Origami Sample Preparation

For enclosed chamber preparation, a flow chamber with an inner volume of 20 µL was formed as described in ref. [8] using a plasma cleaned coverslip (no. 1.5, 18 × 18 mm2, 0.17 mm thick) placed on a plasma cleaned glass slide (3 × 1 in. [11] 1 mm thick) using two strips of double‐sided tape (Scotch, cat. no. 665D). First, 20 µL of biotin‐labeled bovine albumin (1 mg mL^−1^, dissolved in Buffer A) was flown into the chamber and incubated for 2 min. Then the chamber was washed using 40 µL of Buffer A. Second, 20 µL of streptavidin (0.5 mg mL^−1^, dissolved in Buffer A) was then flown through the chamber and incubated for 2 min. Next, the chamber was washed with 40 µL of Buffer A and subsequently with 40 µL of Buffer B. Then, 20 µL of single dye origami structures (1:100–200 dilution in buffer B from folded stock) were flushed into the chamber and incubated for 5 min. Afterwards, the chamber was washed with 40 µL of Buffer B again. Finally, the Buffer C with the oxygen scavenging & triplet state quenching systems was flown into the chamber.

Ibidi 8‐well slides were prepared as follows. A 10 µL drop of biotin labeled bovine albumin (1 mg mL^−1^, dissolved in buffer A) was placed at the chamber center and incubated for 2 min and aspirated. The chamber was then washed with 200 µL of buffer A, aspirated, and then a 10 µL drop streptavidin (0.5 mg mL^−1^, dissolved in buffer A) was placed at the chamber center and incubated for 2 min. After aspirating and washing with 200 µL of buffer A and subsequently with 200 µL of buffer B, a 10 µL of DNA origami (1:100–200 dilution in buffer B from folded stock) was placed at the chamber center and incubated for 5 min. Next, the chamber was washed 2× with 200 µL of Buffer B. Finally, Buffer C with the oxygen scavenging & triplet state quenching systems and imager strand (final concentration of ∼235 pM) was added for DNA‐PAINT imaging.

### 4.7 Conjugation of Secondary Antibodies With Docking Strands

DNA antibody conjugations were performed as previously described [23] in 0.5‐mL Amino Ultra Centrifugal Filters with 50 kDa molecular weight cutoffs with DBCO‐sulfo‐NHS ester cross‐linker, which was dissolved at 20 mM DMSO and stored in single‐use aliquots at −80°C. This cross‐linker links azide‐functionalized DNA oligonucleotides to surface‐exposed lysine residues. Azide‐functionalized DNA oligonucleotides were stored in 1 mM deionized water. Critically, all antibodies were ordered carrier‐free, as common preservatives such as bovine serum albumin and sodium azide interfere with the conjugation reaction. First, 500 µL PBS was added to the Amicon filters, which were centrifuged for 5 min at 10 000 rcf. After wetting the filters, 25 µg antibody was added and washed twice with PBS. For each wash, PBS was added to a total volume of 500 µL, and the filters were centrifuged for 5 min at 10 000 rcf. If after the second spin, the total volume remaining in each filter was greater than 100 µL, the filters were centrifuged again for 5 min at 10 000 rcf. After the second PBS wash, a 20‐fold molar excess of DBCO‐sulfo‐NHS ester cross‐linker and a 20‐fold molar excess of DNA oligonucleotide were added, and after gentle mixing, each conjugation reaction was incubated in the dark at 4°C overnight. The following day, conjugated antibodies were washed three times with PBS, as described above. To elute the antibody, the filter was inverted in a fresh tube and centrifuged for 2 min at 1500 rcf. The conjugated antibody was transferred to a clean tube and stored at −20°C in antibody storage buffer. Concentrations were measured using the Qubit Protein Assay. DNA‐antibody conjugation was confirmed by comparing unconjugated and conjugated antibodies on NuPage 4%–12% Bis‐Tris protein gels. For each sample, 0.5 µg total protein was added to NuPage LDS Sample Buffer and 50 mM DTT. Protein was denatured at 80°C for 10 min. Gels were run at 75 V for 5 min, then at 180 V for 60 min. Gels were stained with InstantBlue Coomassie Protein Stain for 15 min at room temperature, rinsed with water, and imaged on a Sapphire Biomolecular Imager (Azure Biosystems).

### 4.8 Cell Culture and Plating

HeLa cells (Atcc, CCL‐2) were maintained in DMEM supplemented with 10% fetal bovine serum at 37°C with 5% CO2 and were checked regularly for mycoplasma contamination. For imaging of whole HeLa cells, ∼8K cells were seeded in each well of an Ibidi 18‐well chamber, placed in the incubator overnight and fixed the following day.

### 4.9 Fixation and Labeling for HeLa Cell Imaging

24 h after seeding HeLa cells in Ibidi 18‐well chambers, cells were pre‐fixed in 0.2% GA, 0.25% Triton X‐100 in PBS for 90 s and then, fixed in 2% GA in PBS for 10 min at 37°C. Next, samples were washed 3× in PBS and then, quenched in 0.1% NaBH4 in PBS for 7 min. Samples were then washed 4× in PBS (30s, 60s, 2 × 5 min), and both blocked and permeabilized in 3% BSA and 0.25% Triton X‐100 in PBS at room temperature for 90 min. Primary rat anti‐alpha tubulin monoclonal IgGs antibody (MA1‐80017, Invitrogen) was added at 1:100 in 3% BSA and 0.1% Triton‐X 100 in PBS and incubated overnight at 4°C. The next morning, samples were washed 4× washes in PBS (30s, 60s, 2× 5 min) and DNA‐conjugated secondary anti‐rat polyclonal IgG antibody (A18747, Invitrogen) was added at 1:100 in 3% BSA and 0.1% Triton‐X 100 in PBCS and incubated for 1 h at room temperature. Samples were quickly washed 3× in PBS, incubated with gold particles as fiducial markers (1:20 in PBS) for 5 min, washed again 3× 5 min in PBS before adding Buffer C with the oxygen scavenging & triplet state quenching systems and imager (final concentration of ∼4 pM) for DNA‐PAINT imaging.

### 4.10 Super‐Resolution Microscopy Setup

TIRF and HILO imaging was carried out on a Nikon Ti inverted microscope equipped with a Nikon Ti‐TIRF‐EM Motorized Illuminator, a Nikon LUN‐F Laser Launch with single fiber output (561 nm, 70 mW) and a Lumencore SpectraX LED Illumination unit. The objective‐type TIRF system with an oil immersion objective (Apo TIRF 100×/1.49 DIC N2). DNA‐PAINT experiments were performed using the 560 nm laser line and fluorescence emission was passed through a Chroma ZT 405/488/561/640 multi‐band pass dichroic mirror mounted on a Nikon TIRF filter cube located in the filter cube turret and a Chroma ET 595/50m band pass emission filter located on a Sutter emission filter wheel within the infinity space of the stand before image recording on a line on a sCMOS camera (Andor, Zyla 4.2) mounted to a standard Nikon camera port.

### 4.11 Imaging Conditions

All fluorescence microscopy data was recorded with the sCMOS camera (2048 × 2048 pixels, pixel size: 6.5 µm). Both microscope and camera were operated with the Nikon Elements software at 2 × 2 binning and 1024 × 1024 pixel field‐of‐view. The camera read out rate was set to 200 MHz and the dynamic range to 16 bit. Laser power for imaging single dye origami structures was 0.1%, and 20‐nm grids and microtubules was 35% (10 mW).

### 4.12 Image Analysis

All DNA‐PAINT imaging data was processed and reconstructed using the Picasso software suite. Briefly, a standard single molecule localization algorithm was applied to the raw SD and 20 nm grid origami image stacks to generate a pointillist super‐resolution reconstruction1. For all surface‐immobilized SD origami experiments, only fluorescent spots that appeared within the first five frames were localized. When measuring signal duration, interruptions of up to two frames were permitted without ending the trace. Following localization, the 20 nm grid DNA origami structures appeared as clusters of localizations after image correlation‐based drift correction1. Several clusters were manually selected using a circular region with a diameter of one camera pixel, and similar clusters were automatically identified. This process was then repeated at higher resolution for individual docking strand localization clusters in the 20 nm grid, using a circular region with a diameter of 0.125 camera pixels.

Kinetic analysis was based on a custom Python package picasso_addon (https://github.com/schwille‐paint/picasso_addon). For SD origami experiments, kinetic paremeters for each pick (e.g., number of localizations, photon counts, t_1/2,_ etc.) were obtained using the spt.immobile_props.main() function. For 20 nm grid origami and microtubule experiments, the docking site sampling rate was determined by counting localizations at each docking strand or within a defined region, respectively, over a specified time interval. Detailed information about the picasso_addon API is available at https://picasso‐addon.readthedocs.io/en/latest/index.html for detailed information about.

### 4.13 Statistical Analysis

Quantitative data are reported as mean ± SEM unless stated otherwise. The value of n denotes replicates. Statistical significance was assessed using a two‐tailed unpaired Student's *t*‐test. Differences were considered significant at *p* < 0.05. Analyses were performed using GraphPad Prism 10.2 (GraphPad Software, San Diego, CA).

## Conflicts of Interest

Potential conflicts of interest for G.M.C. are listed on https://arep.med.harvard.edu/t/. All other authors declare no competing financial interest.

## Supporting information




**Supporting file 1**: smll71997‐sup‐0001‐SuppMat.pdf

## Data Availability

Single‐molecule localization microscopy data generated in this study are openly available in Zenodo at https://www.doi.org/10.5281/zenodo.17566537
