# Supplementary material for: A Simple, Ultrastable, and Cost‐Effective Oxygen‐Scavenging System for Long‐Term DNA‐PAINT Imaging
Source: Small. 2025 Dec 19;22(9):e09092. doi: 10.1002/smll.202509092 (PMC12895217; doi:10.1002/smll.202509092)
Supplement: Supplementary file 1 — Supporting file 1: smll71997‐sup‐0001‐SuppMat.pdf [file SMLL-22-e09092-s001.pdf]

Supplementary Information

A Simple, Ultrastable, and Cost-Effective Oxygen-Scavenging System for Long-Term DNA-PAINT Imaging

Rebecca T. Perelman[a,b], George M. Church[a,c,d,*] and Johannes Stein[a,c,e,*]

[a] Wyss Institute for Biologically Inspired Engineering, Harvard University, Boston, MA, USA
[b] Harvard Biophysics Program, Harvard University, Boston, MA, USA
[c] Department of Genetics, Harvard Medical School, Boston, MA, USA
[d] Harvard-MIT Program in Health Sciences and Technology, Cambridge, MA, USA
[e] Max Planck Institute for Molecular Genetics, Berlin, Germany

*e-mail: gchurch@genetics.med.harvard.edu, stein@molgen.mpg.de

Supplementary Figures

Supplementary Notes

Supplementary Tables

References

Supplementary Figures

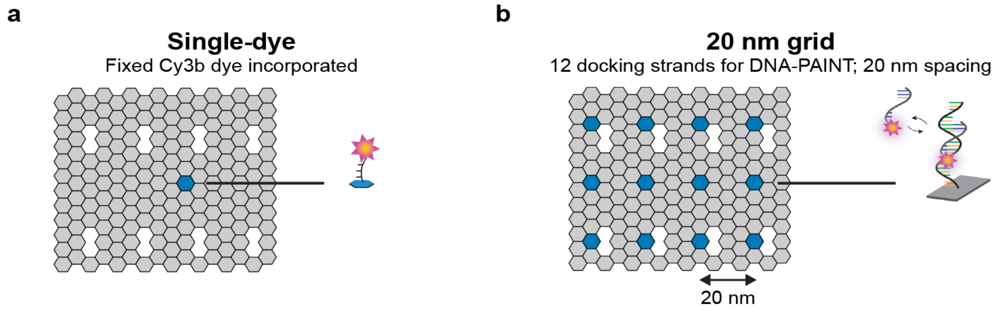

Figure S1 | DNA origami designs. a, Single-dye (SD) DNA origami with fixed Cy3B. **b**, 3×4 20 nm DNA origami grid.

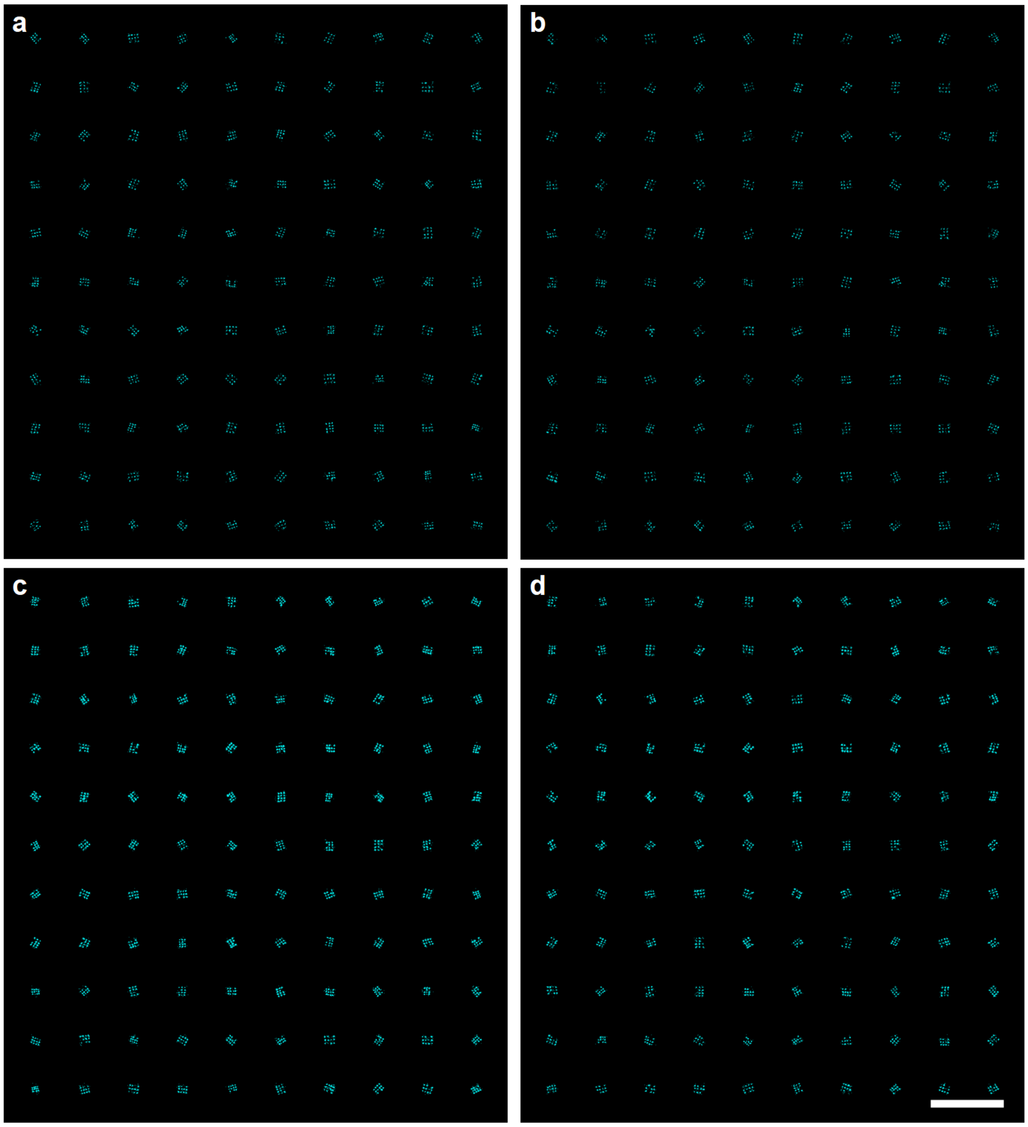

Figure S2 | Individual origami images in SST and PPT immediately after preparation. **a**, Random selection of 110 origami structures in PPT over the first 5,000 frames, displayed in 10×11 grid. **b**, The same 110 origami structures imaged in PPT during the last 5,000 frames. **c**, Random selection of 110 origami structures in SST over the first 5,000 frames, displayed in 10×11 grid. **d**, The same 110 origami structures imaged in SST during the last 5,000 frames. Scale bar: 500 nm. All imaging was performed at the same imager concentration (~235 pM), illumination (10 mW), magnification (100X), exposure time (200 ms), and images were rendered using the same parameters.

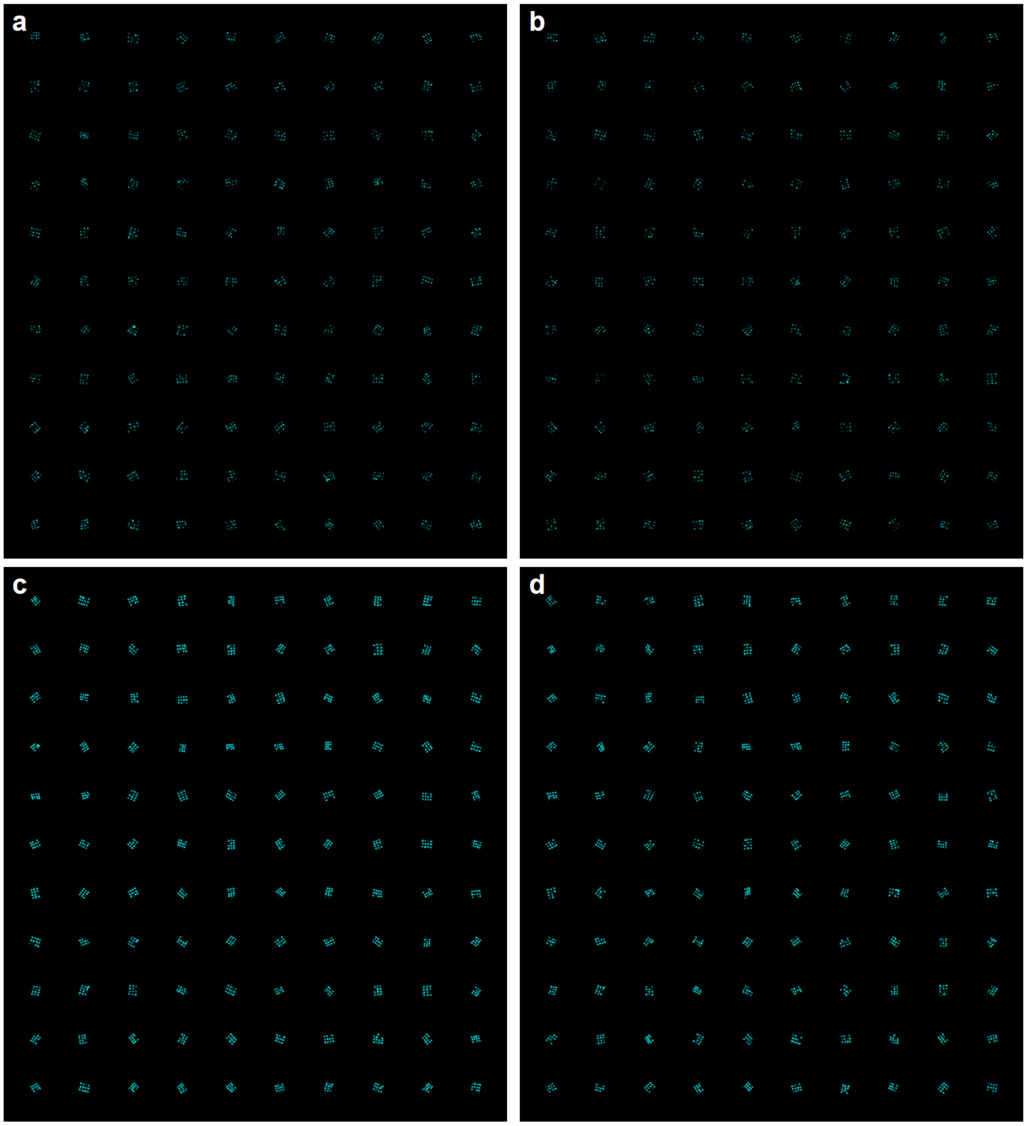

Figure S3 | Individual origami images in SST and PPT 24 hours after preparation. a, Random selection of 110 origami structures in PPT over the first 5,000 frames, displayed in 10×11 grid. **b**, The same 110 origami structures imaged in PPT during the last 5,000 frames. **c**, Random selection of 110 origami structures in SST over the first 5,000 frames, displayed in 10×11 grid. **d**, The same 110 origami structures imaged in SST during the last 5,000 frames. Scale bar: 500 nm. All imaging was performed at the same imager concentration (~235 pM), illumination (10 mW), magnification (100X), exposure time (200 ms), and images were rendered using the same parameters.

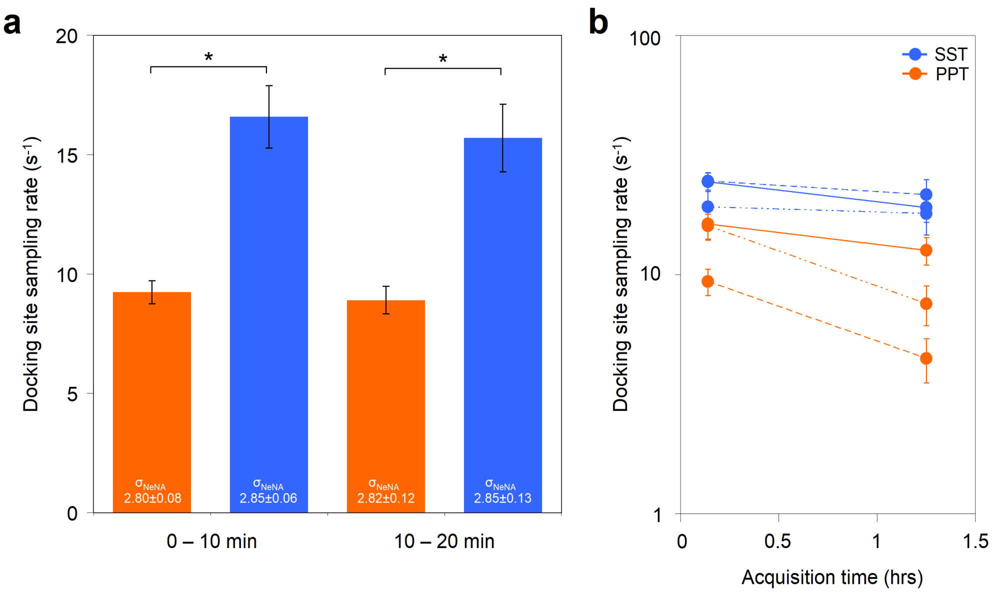

Figure S4 | Docking site sampling rate in SST and PPT. a, Average docking site sampling rate (s^{-1}) measured across three wells imaged for 5,000 frames, with analysis performed in two segments of 2,500 frames each. Results compare PPT (orange) and SST (blue). Data are presented as means ±SEM (n=3). * $p < 0.05$, two-tailed unpaired t-test. σ_{NeNA} values are presented as means ±SD (n=3). **b**, Docking site sampling rate over three successive 1.5-hour imaging sessions for both PPT (orange) and SST (blue): initial imaging immediately after buffer preparation (solid lines), after 24-hour sample storage (dashed lines), and after one month of storage at room-temperature (dotted lines). Data are presented as means ±SEM (n=11).

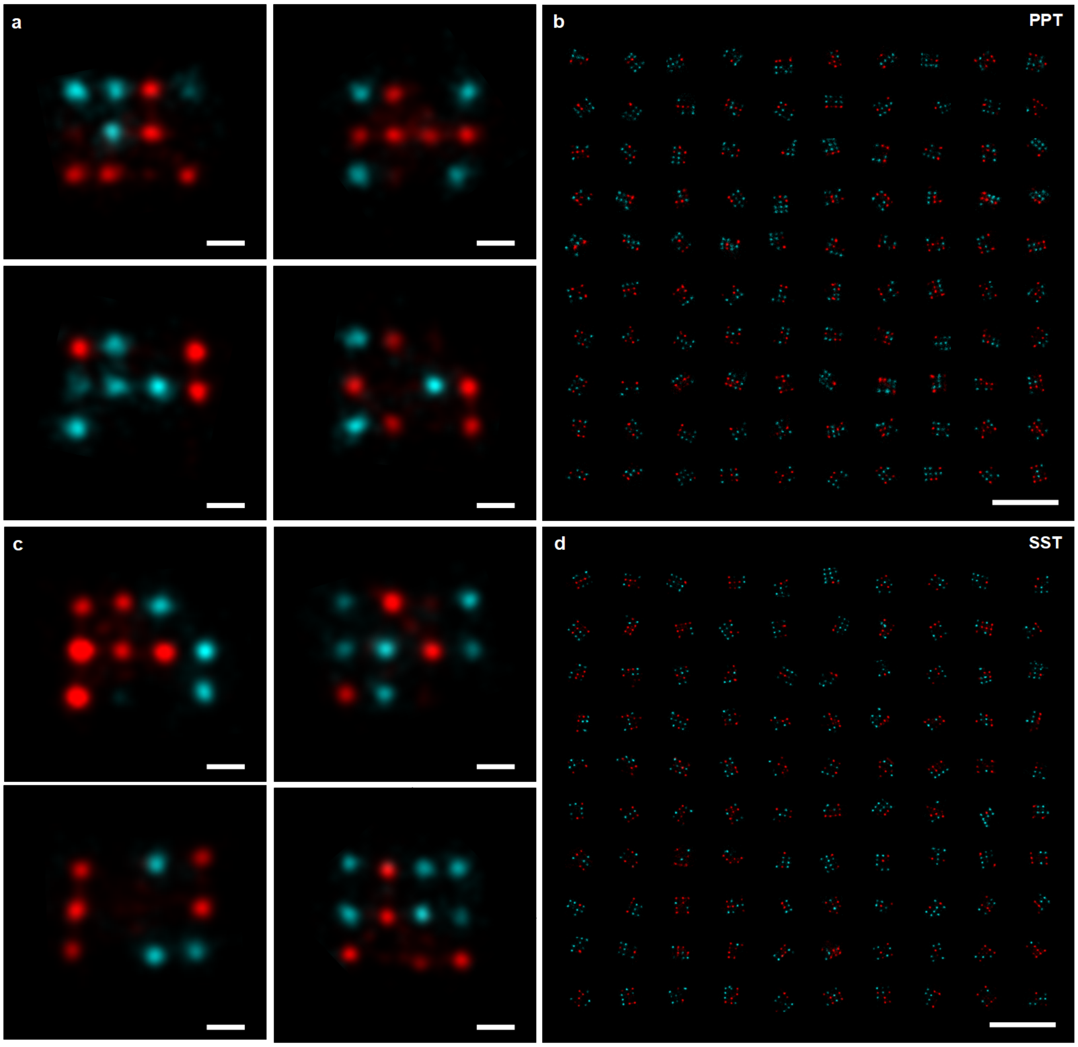

Figure S5 | Exchange-PAINT origami images in SST and PPT. a, Representative Exchange-PAINT images of two docking sites, R3 (cyan) and R4 (red), acquired in PPT, showing no cross-talk between distinct docking and imager strand sequences. Scale bar: 20 nm. **b**, Random selection of 100 origami structures imaged by Exchange-PAINT in PPT, displayed in 10×10 grid. Scale bar: 250 nm. **c**, Representative Exchange-PAINT images of two docking sites, R3 (cyan) and R4 (red), acquired in SST, showing no cross-talk between distinct docking and imager strand sequences. Scale bar: 20 nm. **d**, Random selection of 100 origami structures imaged by Exchange-PAINT in SST, displayed in 10×10 grid. Scale bar: 250 nm. All imaging was performed at the same imager concentration (~1 nM), illumination (10 mW), magnification (100X), exposure time (200 ms), and images were rendered using the same parameters.

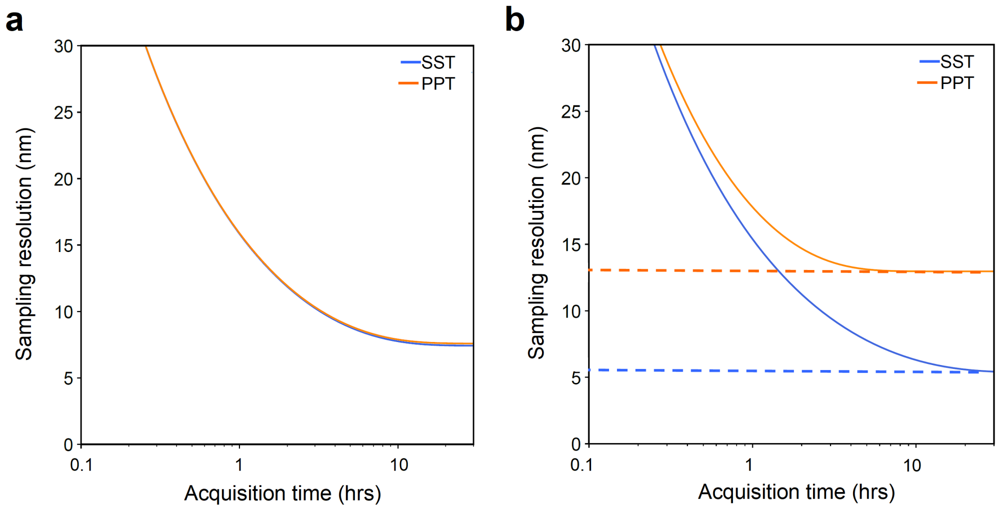

Figure S6 | Docking site sampling resolution in SST and PPT. a, Resolution as a function of acquisition time (*T*), estimated damage rates in SST and PPT immediately after preparation. Under these conditions, the achievable sampling resolution is comparable in both systems, plateauing at approximately 7 nm. **b**, Resolution as a function of acquisition time (T) on logarithmic scale derived from estimated damage rates in SST and PPT buffers, 24 hours after buffer preparation, illustrating how resolution improves through enhanced sampling density. At early time points, the number of accumulated blinks increases linearly; however, over longer durations, docking strand damage limits the number of independent localizations, causing resolution to plateau (dashed lines). SST achieves more than a twofold improvement in resolution compared to PPT.

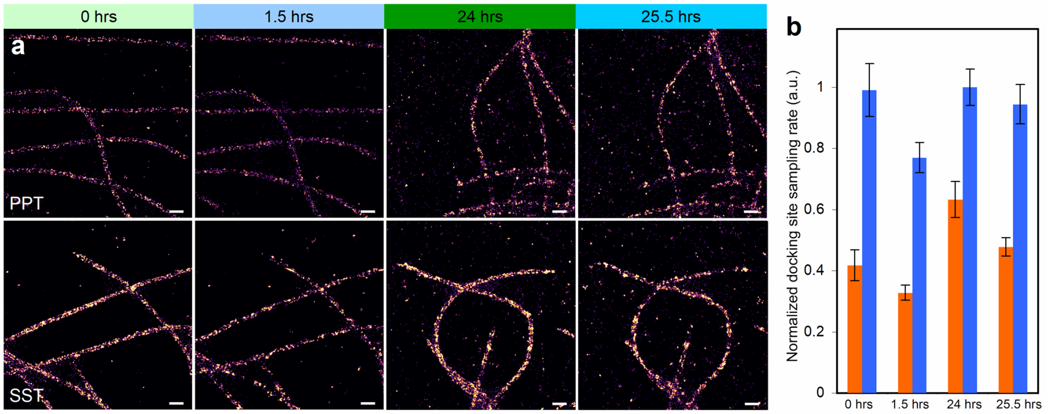

Figure S7 | Comparison of microtubule image quality in PPT and SST. a, Magnified sections highlighting image quality in PPT (top) and SST (bottom) over two 1.5-hour imaging sessions separated by 24 hours of idle time. Scale bars: 250 nm. **b**, Due to the lack of single docking strand resolution, 12 microtubule segments per FOV (130 × 130 μm²) from Figure 3 were selected for analysis, each 4 camera pixels (~520 nm) in length. Docking site sampling rates were quantified for each segment under PPT (orange) and SST (blue) conditions, and normalized to the highest number of localizations observed in any one image. Data are presented as means ±SEM (n=12).

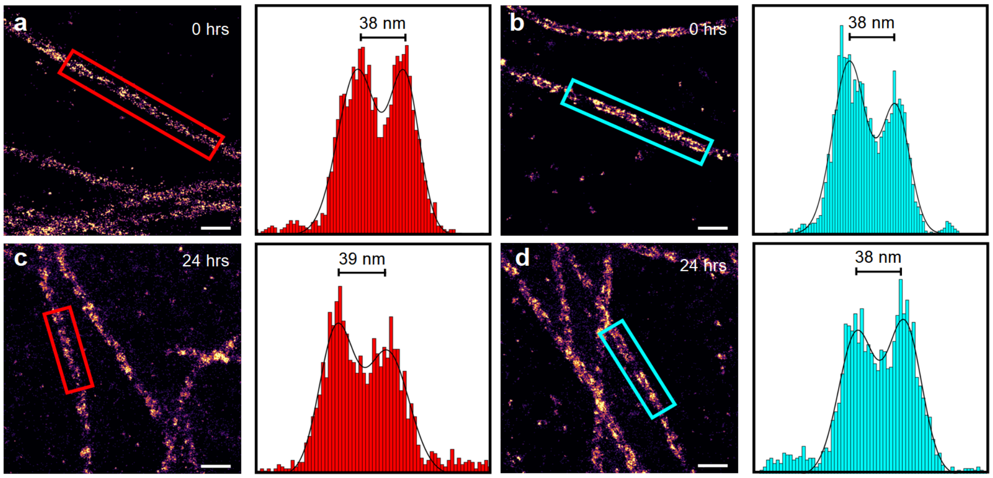

Figure S8 | Microtubule cross-sectional fluorescence intensity profiles. a-d, Microtubule profile analysis comparing PPT (red) and SST (cyan). Left panels: Sections of microtubules with rectangles indicating regions where intensity profiles were extracted. Right panels: Corresponding intensity profiles showing two distinct peaks from labeled microtubule walls projected onto a plane. A double Gaussian fit was applied to determine peak-to-peak distances. Scale bars: 250 nm.

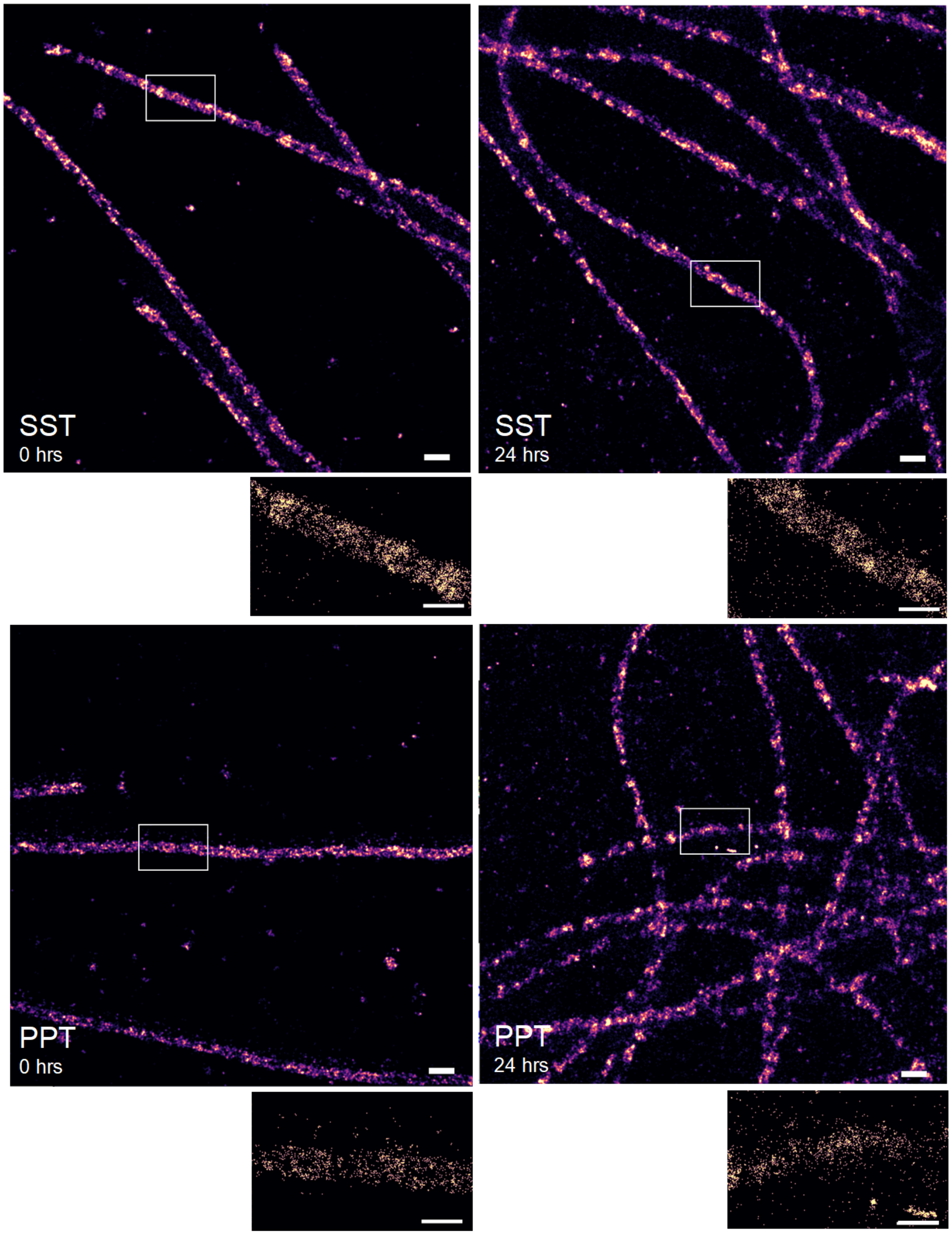

Figure S9 | Microtubule sections at different magnifications. Microtubule regions of interest (ROI) at high magnification (indicated by white boxes and shown below the corresponding low magnification images) highlight individual localizations for each condition. In these small ROIs, the expected double-line profile can be difficult to resolve due to variability in localization counts, likely resulting from differences in labeling efficiency or geometry. In contrast, the pattern becomes more visually apparent over longer microtubule stretches, as seen in the low-magnification images. Scale bars: 200 nm (low magnification), 100 nm (high magnification).

Supplementary Notes

Supplementary Note 1. In order to achieve the highest possible image resolution, DNA-PAINT requires both high per-blink localization precision and high sampling density of distinct events. In practice, DNA-PAINT experiments often last from minutes to many hours to accumulate sufficient sampling density. During these long acquisitions, docking strands can be irreversibly damaged by ROS generated under illumination, reducing the number of docking sites and slowing down further sampling. Photostability thus imposes an ultimate upper limit on resolution, even a system with perfect per-blink precision will fail to resolve fine features if sites are lost before blinking. Localization precision, how accurately the position of a single blink can be estimated, is the equation for the variance of a fitted Gaussian centroid on a pixelated camera[1] and is inversely proportional to the square root of the number of photons, *N*. In DNA-PAINT, however, each docking strand can blink many times, and statistical averaging of *M* independent localizations from the same site reduces uncertainty by a factor of $\sqrt{M}$. In principle, such averaging can push precision into the sub-nanometer range, provided the camera, optics, and sample remain stable.

While high per-blink precision can, in theory, reach the sub-nanometer regime with sufficient photon counts, practical limitations, including docking strand length, linker flexibility, and biological variability, place additional constraints on achievable resolution. These effects are particularly important when imaging unknown biological structures, where docking site positions are not predetermined. In such cases, achieving high sampling density becomes essential to resolve fine structural details.

However, when imaging an unknown biological structure, one cannot assume knowledge of docking strand positions. If docking sites are arranged in a dense pattern to label a structure of interest, achieving a high distinct localization density across the region becomes critical. The Nyquist-Shannon sampling theorem is a common method for linking image resolution to molecular sampling density[2,3]. In two dimensions, the density of distinct localizations satisfies

$$d_{\text{Nyquist}} = \frac{2}{\sqrt{\rho}} \tag{1}$$

where ρ is the density of localizations per unit area. If each site produces *M* independent localizations, the effective sampling density of localizations in the image is $\rho_{loc} = \rho_0 M$, where ρ_0 is the density of potential binding locations, in other words docking strands, per unit area. This gives

$$d_{\text{Nyquist}} = \frac{2}{\sqrt{\rho_0 M}} \tag{2}$$

Thus even an imaging system capable of sub-nanometer photon-limited precision will only be able to resolve features of size d_{Nyquist} if the sampling density is sufficiently high. A reduction in available docking sites over time directly constrains the highest resolvable spatial frequencies.

In order to estimate how many blinks per site accumulate over a continuous acquisition time, *T*, each docking strand can be described as undergoing three competing first-order processes: binding of an imager at a rate $k_{\text{on}}[c]$ (units s⁻¹, proportional to imager concentration *c*), unbinding at a rate k_{off} (s⁻¹), and irreversible damage at a rate k_{dmg} (s⁻¹)[4]. The only process that reduces site availability is damage. Let *S(t)* denote the probability that a site remains available at time *t*

$$\frac{dS}{dt} = -k_{\text{dmg}} \Rightarrow S(t) = e^{-k_{\text{dmg}} t} \tag{3}$$

The instantaneous rate of new blink events is $k_{\text{on}}[c]S(t)$. Integrating from time zero to *T* yields the total cumulative number of blinks per site:

$$M(t)=\int_0^T k_{\mathrm{on}}[c]e^{-k_{\mathrm{dmg}}t}=\frac{k_{\mathrm{on}}[c]}{k_{\mathrm{dmg}}}\left(1-e^{-k_{\mathrm{dmg}}t}\right) \tag{4}$$

At short times $\left(k_{\mathrm{dmg}}T \ll 1\right)$, blink count grows linearly as $k_{\mathrm{on}}[c]S(t)$ and at long times $\left(k_{\mathrm{dmg}}T \gg 1\right)$, blink accumulation saturates at $k_{\mathrm{on}}[c]/k_{\mathrm{dmg}}$, due to the progressive loss of active docking sites through irreversible damage.

The damage rate k_{dmg}, defines the characteristic half-life for docking strand survival as

$$t_{1/2}=\frac{\ln 2}{k_{\mathrm{dmg}}} \tag{5}$$

We developed a simple predictive model using damage rates estimated from the known docking site positions in the 20 nm grid origami. Immediately after buffer preparation, both PPT and SST produced comparable damage rates and predicted sampling-limited resolution of approximately 7 nm (Figure S4c). However, this estimate is based on a 1.5-hour acquisition prior to PPT degradation and likely overstates resolution achievable in longer experiments. To reflect more realistic usage, we focused our analysis on the 24-hour sample storage condition, which offers a clearer comparison of long-term OSS performance.

Based on our measurements of docking site sampling rates, we estimated damage rates of approximately 2.1×10^{-4} s^{-1} (half-life ~55 minutes) for PPT, and a significantly lower rate of 3.6×10^{-5} s^{-1} (half-life ~5.4 hours) for SST. Using these two regimes, we modeled the evolution of sampling resolution over a 24-hour period.

To calculate Nyquist-limited resolution over any acquisition duration, we substitute Equation (4) into Equation (2), yielding the final expression for the estimated sampling resolution:

$$d_{\mathrm{Nyquist}}(T)=2\sqrt{\frac{k_{\mathrm{dmg}}}{\rho_0 k_{\mathrm{on}}[c]\left(1-e^{-k_{\mathrm{dmg}}t}\right)}} \tag{6}$$

In PPT, resolution gains diminish after about one hour and plateau at approximately 13 nm. In contrast, SST can, in principle, achieve resolutions below 6 nm with multi-hour acquisitions (Figure S4d). Overall, DNA-PAINT image acquisitions using SST are expected to achieve more than a twofold improvement in resolution when imaging durations are sufficiently extended.

Supplementary Tables

Supplementary Table 1 | List of core staples. The core origami staple strand sequences were exported in 96-well plate format using Picasso Design[1] and ordered as 96-well plates from IDT at 100 nmole with standard desalting and adjusted to 100mM in IDTE buffer. The empty positions contain place holders for biotinylated staple oligos which were ordered separately (sequences of

biotinylated oligos can be found in **Supplementary Table 2**). Note that the first number indicates the plate number (i.e. 1A1 = position A1 on plate 1).

Plate/ Position	Name	Sequence
**Plate 1**		
1A1	21[32]23[31]BLK	TTTTCACTCAAAGGGCGAAAAACCATCACC
1A2	19[32]21[31]BLK	GTCGACTTCGGCCAACGCGCGGGGTTTTTC
1A3	17[32]19[31]BLK	TGCATCTTTCCCAGTCACGACGGCCTGCAG
1A4	15[32]17[31]BLK	TAATCAGCGGATTGACCGTAATCGTAACCG
1A5	13[32]15[31]BLK	AACGCAAAATCGATGAACGGTACCGGTTGA
1A6	11[32]13[31]BLK	AACAGTTTTGTACCAAAAACATTTTATTTC
1A7	9[32]11[31]BLK	TTTACCCCAACATGTTTTAAATTTCCATAT
1A8	7[32]9[31]BLK	TTTAGGACAAATGCTTTAAACAATCAGGTC
1A9	5[32]7[31]BLK	CATCAAGTAAAACGAACTAACGAGTTGAGA
1A10	3[32]5[31]BLK	AATACGTTTGAAAGAGGACAGACTGACCTT
1A11	1[32]3[31]BLK	AGGCTCCAGAGGCTTTGAGGACACGGGTAA
1A12	0[47]1[31]BLK	AGAAAGGAACAACTAAAGGAATTCAAAAAAA
1B1	23[32]22[48]BLK	CAAATCAAGTTTTTTGGGGTCGAAACGTGGA
1B2	22[47]20[48]BLK	CTCCAACGCAGTGAGACGGGCAACCAGCTGCA
1B3	20[47]18[48]BLK	TTAATGAACTAGAGGATCCCCGGGGGGTAACG
1B4	18[47]16[48]BLK	CCAGGGTTGCCAGTTTGAGGGGACCCGTGGGA
1B5	16[47]14[48]BLK	ACAAACGGAAAAGCCCCAAAAACACTGGAGCA
1B6	14[47]12[48]BLK	AACAAGAGGGATAAAAATTTTTAGCATAAAGC
1B7	12[47]10[48]BLK	TAAATCGGGATTCCCAATTCTGCGATATAATG
1B8	10[47]8[48]BLK	CTGTAGCTTGACTATTATAGTCAGTTCATTGA
1B9	8[47]6[48]BLK	ATCCCCCTATACCACATTCAACTAGAAAAATC
1B10	6[47]4[48]BLK	TACGTTAAAGTAATCTTGACAAGAACCGAACT
1B11	4[47]2[48]BLK	GACCAACTAATGCCACTACGAAGGGGGTAGCA
1B12	2[47]0[48]BLK	ACGGCTACAAAAGGAGCCTTTAATGTGAGAAT
1C1	21[56]23[63]BLK	AGCTGATTGCCCTTCAGAGTCCACTATTAAAGGGTGCCGT
1C2		
1C3		
1C4	15[64]18[64]BLK	GTATAAGCCAACCCGTCGGATTCTGACGACAGTATCGGCCGCAAGGCG
1C5	13[64]15[63]BLK	TATATTTTGTCATTGCCTGAGAGTGGAAGATT
1C6	11[64]13[63]BLK	GATTTAGTCAATAAAGCCTCAGAGAACCCTCA
1C7	9[64]11[63]BLK	CGGATTGCAGAGCTTAATTGCTGAAACGAGTA

1C8	7[56]9[63]BLK	ATGCAGATACATAACGGGAATCGTCATAAATAAAGCAAAG
1C9		
1C10		
1C11	1[64]4[64]BLK	TTTATCAGGACAGCATCGGAACGACACCAACCTAAAACGAGGTCAATC
1C12	0[79]1[63]BLK	ACAACTTTCAACAGTTTCAGCGGATGTATCGG
1D1	23[64]22[80]BLK	AAAGCACTAAATCGGAACCCTAATCCAGTT
1D2	22[79]20[80]BLK	TGGAACAACCGCCTGGCCCTGAGGCCCGCT
1D3	20[79]18[80]BLK	TTCCAGTCGTAATCATGGTCATAAAAGGGG
1D4	18[79]16[80]BLK	GATGTGCTTCAGGAAGATCGCACAATGTGA
1D5	16[79]14[80]BLK	GCGAGTAAAAATATTTAAATTGTTACAAAG
1D6	14[79]12[80]BLK	GCTATCAGAAATGCAATGCCTGAATTAGCA
1D7	12[79]10[80]BLK	AAATTAAGTTGACCATTAGATACTTTTGCG
1D8	10[79]8[80]BLK	GATGGCTTATCAAAAAGATTAAGAGCGTCC
1D9	8[79]6[80]BLK	AATACTGCCCAAAAGGAATTACGTGGCTCA
1D10	6[79]4[80]BLK	TTATACCACCAAATCAACGTAACGAACGAG
1D11	4[79]2[80]BLK	GCGCAGACAAGAGGCAAAAGAATCCCTCAG
1D12	2[79]0[80]BLK	CAGCGAAACTTGCTTTCGAGGTGTTGCTAA
1E1	21[96]23[95]BLK	AGCAAGCGTAGGGTTGAGTGTTGTAGGGAGCC
1E2	19[96]21[95]BLK	CTGTGTGATTGCGTTGCGCTCACTAGAGTTGC
1E3	17[96]19[95]BLK	GCTTTCCGATTACGCCAGCTGGCGGCTGTTTC
1E4	15[96]17[95]BLK	ATATTTTGGCTTTCATCAACATTATCCAGCCA
1E5	13[96]15[95]BLK	TAGGTAAACTATTTTTGAGAGATCAAACGTTA
1E6	11[96]13[95]BLK	AATGGTCAACAGGCAAGGCAAAGAGTAATGTG
1E7	9[96]11[95]BLK	CGAAAGACTTTGATAAGAGGTCATATTTCGCA
1E8	7[96]9[95]BLK	TAAGAGCAAATGTTTAGACTGGATAGGAAGCC
1E9	5[96]7[95]BLK	TCATTCAGATGCGATTTTAAGAACAGGCATAG
1E10	3[96]5[95]BLK	ACACTCATCCATGTTACTTAGCCGAAAGCTGC
1E11	1[96]3[95]BLK	AAACAGCTTTTTGCGGGATCGTCAACACTAAA
1E12	0[111]1[95]BLK	TAAATGAATTTTCTGTATGGGATTAATTTCTT
1F1	23[96]22[112]BLK	CCCGATTTAGAGCTTGACGGGGAAAAAGAATA
1F2	22[111]20[112]BLK	GCCCGAGAGTCCACGCTGGTTTGCAGCTAACT
1F3	20[111]18[112]BLK	CACATTAAAATTGTTATCCGCTCATGCGGGCC
1F4	18[111]16[112]BLK	TCTTCGCTGCACCGCTTCTGGTGCGGCCTTCC
1F5	16[111]14[112]BLK	TGTAGCCATTAAAATTCGCATTAAATGCCGGA
1F6	14[111]12[112]BLK	GAGGGTAGGATTCAAAAGGGTGAGACATCCAA
1F7	12[111]10[112]BLK	TAAATCATATAACCTGTTTAGCTAACCTTTAA

1F8	10[111]8[112]BLK	TTGCTCCTTTCAAATATCGCGTTTGAGGGGGT
1F9	8[111]6[112]BLK	AATAGTAAACACTATCATAACCCTCATTGTGA
1F10	6[111]4[112]BLK	ATTACCTTTGAATAAGGCTTGCCCAAATCCGC
1F11	4[111]2[112]BLK	GACCTGCTCTTTGACCCCCAGCGAGGGAGTTA
1F12	2[111]0[112]BLK	AAGGCCGCTGATACCGATAGTTGCGACGTTAG
1G1	21[120]23[127]BLK	CCCAGCAGGCGAAAAATCCCTTATAAATCAAGCCGGCG
1G2		
1G3		
1G4	15[128]18[128]BLK	TAAATCAAAATAATTCGCGTCTCGGAAACCAGGCAAAGGGAAGG
1G5	13[128]15[127]BLK	GAGACAGCTAGCTGATAAATTAATTTTTGT
1G6	11[128]13[127]BLK	TTTGGGGATAGTAGTAGCATTAAAAGGCCG
1G7	9[128]11[127]BLK	GCTTCAATCAGGATTAGAGAGTTATTTTCA
1G8	7[120]9[127]BLK	CGTTTACCAGACGACAAAGAAGTTTTGCCATAATTCGA
1G9		
1G10		
1G11	1[128]4[128]BLK	TGACAACTCGCTGAGGCTTGCATTATACCAAGCGCGATGATAAA
1G12	0[143]1[127]BLK	TCTAAAGTTTTGTCGTCTTTCCAGCCGACAA
1H1	21[160]22[144]BLK	TCAATATCGAACCTCAAATATCAATTCCGAAA
1H2	19[160]20[144]BLK	GCAATTCACATATTCCTGATTATCAAAGTGTA
1H3	17[160]18[144]BLK	AGAAAACAAAGAAGATGATGAAACAGGCTGCG
1H4	15[160]16[144]BLK	ATCGCAAGTATGTAAATGCTGATGATAGGAAC
1H5	13[160]14[144]BLK	GTAATAAGTTAGGCAGAGGCATTTATGATATT
1H6	11[160]12[144]BLK	CCAATAGCTCATCGTAGGAATCATGGCATCAA
1H7	9[160]10[144]BLK	AGAGAGAAAAAAATGAAAATAGCAAGCAAACT
1H8	7[160]8[144]BLK	TTATTACGAAGAACTGGCATGATTGCGAGAGG
1H9	5[160]6[144]BLK	GCAAGGCCTCACCAGTAGCACCATGGGCTTGA
1H10	3[160]4[144]BLK	TTGACAGGCCACCACCAGAGCCGCGATTTGTA
1H11	1[160]2[144]BLK	TTAGGATTGGCTGAGACTCCTCAATAACCGAT
1H12	0[175]0[144]BLK	TCCACAGACAGCCCTCATAGTTAGCGTAACGA
**Plate 2**		
2A1	23[128]23[159]BLK	AACGTGGCGAGAAAGGAAGGGAAACCAGTAA
2A2	22[143]21[159]BLK	TCGGCAAATCCTGTTTGATGGTGGACCCTCAA
2A3	20[143]19[159]BLK	AAGCCTGGTACGAGCCGGAAGCATAGATGATG
2A4	18[143]17[159]BLK	CAACTGTTGCGCCATTCGCCATTCAAACATCA
2A5	16[143]15[159]BLK	GCCATCAAGCTCATTTTTTAACCACAAATCCA
2A6	14[143]13[159]BLK	CAACCGTTTCAAATCACCATCAATTCGAGCCA

2A7	12[143]11[159]BLK	TTCTACTACGCGAGCTGAAAAGGTTACCGCGC
2A8	10[143]9[159]BLK	CCAACAGGAGCGAACCAGACCGGAGCCTTTAC
2A9	8[143]7[159]BLK	CTTTTGCAGATAAAAACCAAAATAAAGACTCC
2A10	6[143]5[159]BLK	GATGGTTTGAACGAGTAGTAAATTTACCATTA
2A11	4[143]3[159]BLK	TCATCGCCAACAAAGTACAACGGACGCCAGCA
2A12	2[143]1[159]BLK	ATATTCGGAACCATCGCCCACGCAGAGAAGGA
2B1	23[160]22[176]BLK	TAAAAGGGACATTCTGGCCAACAAAGCATC
2B2	22[175]20[176]BLK	ACCTTGCTTGGTCAGTTGGCAAAGAGCGGA
2B3	20[175]18[176]BLK	ATTATCATTCAATATAATCCTGACAATTAC
2B4	18[175]16[176]BLK	CTGAGCAAAAATTAATTACATTTTGGGTTA
2B5	16[175]14[176]BLK	TATAACTAACAAAGAACGCGAGAACGCCAA
2B6	14[175]12[176]BLK	CATGTAATAGAATATAAAGTACCAAGCCGT
2B7	12[175]10[176]BLK	TTTTATTTAAGCAAATCAGATATTTTTTGT
2B8	10[175]8[176]BLK	TTAACGTCTAACATAAAAACAGGTAACGGA
2B9	8[175]6[176]BLK	ATACCCAACAGTATGTTAGCAAATTAGAGC
2B10	6[175]4[176]BLK	CAGCAAAAGGAAACGTCACCAATGAGCCGC
2B11	4[175]2[176]BLK	CACCAGAAAGGTTGAGGCAGGTCATGAAAG
2B12	2[175]0[176]BLK	TATTAAGAAGCGGGGTTTTGCTCGTAGCAT
2C1	21[184]23[191]BLK	TCAACAGTTGAAAGGAGCAAATGAAAAATCTAGAGATAGA
2C2		
2C3		
2C4	15[192]18[192]BLK	TCAAATATAACCTCCGGCTTAGGTAACAATTTCATTTGAAGGCGAATT
2C5	13[192]15[191]BLK	GTAAAGTAATCGCCATATTTAACAAAACTTTT
2C6	11[192]13[191]BLK	TATCCGGTCTCATCGAGAACAAGCGACAAAAG
2C7	9[192]11[191]BLK	TTAGACGGCCAAATAAGAAACGATAGAAGGCT
2C8	7[184]9[191]BLK	CGTAGAAAATACATACCGAGGAAACGCAATAAGAAGCGCA
2C9		
2C10		
2C11	1[192]4[192]BLK	GCGGATAACCTATTATTCTGAAACAGACGATTGGCCTTGAAGAGCCAC
2C12	0[207]1[191]BLK	TCACCAGTACAAACTACAACGCCTAGTACCAG
2D1	23[192]22[208]BLK	ACCCTTCTGACCTGAAAGCGTAAGACGCTGAG
2D2	22[207]20[208]BLK	AGCCAGCAATTGAGGAAGGTTATCATCATTTT
2D3	20[207]18[208]BLK	GCGGAACATCTGAATAATGGAAGGTACAAAAT
2D4	18[207]16[208]BLK	CGCGCAGATTACCTTTTTTAATGGGAGAGACT
2D5	16[207]14[208]BLK	ACCTTTTTATTTTAGTTAATTTCATAGGGCTT
2D6	14[207]12[208]BLK	AATTGAGAATTCTGTCCAGACGACTAAACCAA

2D7	12[207]10[208]BLK	GTACCGCAATTCTAAGAACGCGAGTATTATTT
2D8	10[207]8[208]BLK	ATCCCAATGAGAATTAACTGAACAGTTACCAG
2D9	8[207]6[208]BLK	AAGGAAACATAAAGGTGGCAACATTATCACCG
2D10	6[207]4[208]BLK	TCACCGACGCACCGTAATCAGTAGCAGAACCG
2D11	4[207]2[208]BLK	CCACCCTCTATTCACAAACAAATACCTGCCTA
2D12	2[207]0[208]BLK	TTTCGGAAGTGCCGTCGAGAGGGTGAGTTTCG
2E1	21[224]23[223]BLK	CTTTAGGGCCTGCAACAGTGCCAATACGTG
2E2	19[224]21[223]BLK	CTACCATAGTTTGAGTAACATTTAAAATAT
2E3	17[224]19[223]BLK	CATAAATCTTTGAATACCAAGTGTTAGAAC
2E4	15[224]17[223]BLK	CCTAAATCAAAATCATAGGTCTAAACAGTA
2E5	13[224]15[223]BLK	ACAACATGCCAACGCTCAACAGTCTTCTGA
2E6	11[224]13[223]BLK	GCGAACCTCCAAGAACGGGTATGACAATAA
2E7	9[224]11[223]BLK	AAAGTCACAAAATAAACAGCCAGCGTTTTA
2E8	7[224]9[223]BLK	AACGCAAAGATAGCCGAACAAACCCTGAAC
2E9	5[224]7[223]BLK	TCAAGTTTCATTAAAGGTGAATATAAAAGA
2E10	3[224]5[223]BLK	TTAAAGCCAGAGCCGCCACCCTCGACAGAA
2E11	1[224]3[223]BLK	GTATAGCAAACAGTTAATGCCCAATCCTCA
2E12	0[239]1[223]BLK	AGGAACCCATGTACCGTAACACTTGATATAA
2F1	23[224]22[240]BLK	GCACAGACAATATTTTTGAATGGGGTCAGTA
2F2	22[239]20[240]BLK	TTAACACCAGCACTAACAACTAATCGTTATTA
2F3	20[239]18[240]BLK	ATTTTAAAATCAAAATTATTTGCACGGATTCG
2F4	18[239]16[240]BLK	CCTGATTGCAATATATGTGAGTGATCAATAGT
2F5	16[239]14[240]BLK	GAATTTATTTAATGGTTTGAAATATTCTTACC
2F6	14[239]12[240]BLK	AGTATAAAGTTCAGCTAATGCAGATGTCTTTC
2F7	12[239]10[240]BLK	CTTATCATTCCCGACTTGCGGGAGCCTAATTT
2F8	10[239]8[240]BLK	GCCAGTTAGAGGGTAATTGAGCGCTTTAAGAA
2F9	8[239]6[240]BLK	AAGTAAGCAGACACCACGGAATAATATTGACG
2F10	6[239]4[240]BLK	GAAATTATTGCCTTTAGCGTCAGACCGGAACC
2F11	4[239]2[240]BLK	GCCTCCCTCAGAATGGAAAGCGCAGTAACAGT
2F12	2[239]0[240]BLK	GCCCGTATCCGGAATAGGTGTATCAGCCCAAT
2G1	21[248]23[255]BLK	AGATTAGAGCCGTCAAAAAACAGAGGTGAGGCCTATTAGT
2G2		
2G3		
2G4	15[256]18[256]BLK	GTGATAAAAAGACGCTGAGAAGAGATAACCTTGCTTCTGTTCGGGAGA
2G5	13[256]15[255]BLK	GTTTATCAATATGCGTTATACAAACCGACCGT
2G6	11[256]13[255]BLK	GCCTTAAACCAATCAATAATCGGCACGCGCCT

2G7	9[256]11[255]BLK	GAGAGATAGAGCGTCTTTCCAGAGGTTTTGAA
2G8	7[248]9[255]BLK	GTTTATTTTGTCACAATCTTACCGAAGCCCTTTAATATCA
2G9		
2G10		
2G11	1[256]4[256]BLK	CAGGAGGTGGGGTCAGTGCCTTGAGTCTCTGAATTTACCGGGAACCAG
2G12	0[271]1[255]BLK	CCACCCTCATTTTCAGGGATAGCAACCGTACT
2H1	23[256]22[272]BLK	CTTTAATGCGCGAACTGATAGCCCCACCAG
2H2	22[271]20[272]BLK	CAGAAGATTAGATAATACATTTGTCGACAA
2H3	20[271]18[272]BLK	CTCGTATTAGAAATTGCGTAGATACAGTAC
2H4	18[271]16[272]BLK	CTTTTACAAAATCGTCGCTATTAGCGATAG
2H5	16[271]14[272]BLK	CTTAGATTTAAGGCGTTAAATAAAGCCTGT
2H6	14[271]12[272]BLK	TTAGTATCACAATAGATAAGTCCACGAGCA
2H7	12[271]10[272]BLK	TGTAGAAATCAAGATTAGTTGCTCTTACCA
2H8	10[271]8[272]BLK	ACGCTAACACCCACAAGAATTGAAAATAGC
2H9	8[271]6[272]BLK	AATAGCTATCAATAGAAAATTCAACATTCA
2H10	6[271]4[272]BLK	ACCGATTGTCGGCATTTTCGGTCATAATCA
2H11	4[271]2[272]BLK	AAATCACCTTCCAGTAAGCGTCAGTAATAA
2H12	2[271]0[272]BLK	GTTTTAACTTAGTACCGCCACCCAGAGCCA

Supplementary Table 2 | List of biotinylated staples. Biotinilated staples were ordered from IDT at 100 nmole with standard desalting and adjusted to 100mM in IDTE buffer.

No	Name	Sequence	Mod
1	18[63]20[56]BIOTIN	ATTAAGTTTACCGAGCTCGAATTCGGGAAACCTGTCGTGC	5'-BT
2	4[63]6[56]BIOTIN	ATAAGGGAACCGGATATTCATTACGTCAGGACGTTGGGAA	5'-BT
3	18[127]20[120]BIOTIN	GCGATCGGCAATTCCACACAACAGGTGCCTAATGAGTG	5'-BT
4	4[127]6[120]BIOTIN	TTGTGTCGTGACGAGAAACACCAAATTTCAACTTTAAT	5'-BT
5	18[191]20[184]BIOTIN	ATTCATTTTTGTTTGGATTATACTAAGAAACCACCAGAAG	5'-BT
6	4[191]6[184]BIOTIN	CACCCTCAGAAACCATCGATAGCATTGAGCCATTTGGGAA	5'-BT
7	18[255]20[248]BIOTIN	AACAATAACGTAAAACAGAAATAAAAATCCTTTGCCCGAA	5'-BT
8	4[255]6[248]BIOTIN	AGCCACCACTGTAGCGCGTTTTCAAGGGAGGGAAGGTAAA	5'-BT

Supplementary Table 3 | Functional staple sequences. The following functional oligos were used to replace core staple position in the respective DNA origami design ('SD' – fixed single-dye origami[3] and '20nm' – 20-nm grid with 3x4 pattern of docking strands for DNA-PAINT). The docking strand-extended staples were ordered from IDT at 100nmole with standard desalting at 100mM in IDTE buffer for standard docking strands and the fixed-Cy3b modified staple at 250nmole with HPLC purification and lyophilized.

Short name (docking site length)	Origami ID	Docking strand sequence	Concatenated to following staple positions
5xCTC	20nm	TT CTCCTCCTCCTCCTC	1B3, 2B3, 1F3, 2F3<br>1B7, 2B7, 1F7, 2F7<br>1B11, 2B11, 1F11, 2F11
4T_Cy3b	SD	TTTT-Cy3b	2B7
A20 - adapter	20nm	TT AAGAAAGAAAAGAAGAAAAG	1B3, 2B3, 1F3, 2F3<br>1B7, 2B7, 1F7, 2F7<br>1B11, 2B11, 1F11, 2F11

Supplementary Table 4 | Adapter strand sequences. The following oligos were used for Exchange-PAINT experiments using an intermediate stable binding adapter to install different docking strands onto each A20 DNA origami. The adapter strands were ordered from IDT at 100nmole with standard desalting at 100mM in IDTE buffer.

Short name	Adapter sequence
cA15_tt_7xR3	CTTTTCTTCTTTTCTttCTCTCTCTCTCTCTCTCTC
cA15_tt_7xR4	CTTTTCTTCTTTTCTttACACACACACACACACACA

Supplementary Table 5 | Imager strand sequence. Cy3b-labeled imager strand was ordered from IDT at 250nmole with HPLC purification and lyophilized. The stock solution was adjusted to 100µM and stored at -20 °C. Working aliquots at 1 µM were stored in the dark at 4 °C.

Short name	Imager sequence
Pm2	GAGGAGG-Cy3b
R3	GAGAGAG-Cy3b
R4	TGTGTGT-Cy3b

References

1. R.E. Thompson, D.R. Larson, W.W. Webb. Precise nanometer localization analysis for individual fluorescent probes. *Biophys. J.* **2002**, *82*, 2775-2783.
2. C.E. Shannon. Communication in the presence of noise. *Proc. IRE* **1949**, *37*, 10-21.
3. W.R. Legant, L. Shao, J.B. Grimm, T.A. Brown, D.E. Milkie, B.B. Avants, L.D. Lavis, E. Betzig. High-density three-dimensional localization microscopy across large volumes. *Nat. Methods* **2016**, *13*, 359-365.
4. P. Blumhardt, J. Stein, J. Mücksch, F. Stehr, J. Bauer, R. Jungmann, P. Schwille. Photo-induced depletion of binding sites in DNA-PAINT microscopy. *Molecules* **2018**, *23*, 3165.
